# Effects of environmental stress on mRNA expression levels of seven genes related to oxidative stress and growth in Atlantic salmon *Salmo salar* L. of farmed, hybrid and wild origin

**DOI:** 10.1186/1756-0500-5-672

**Published:** 2012-12-05

**Authors:** Monica F Solberg, Bjørn Olav Kvamme, Frank Nilsen, Kevin A Glover

**Affiliations:** 1 Section of Population Genetics and Ecology, Institute of Marine Research, Nordnes, P.O. Box 1870, N-5817, Bergen, Norway; 2 Department of Biology, University of Bergen, Box 7800, 5020, Bergen, Norway; 3 Section of Health, Institute of Marine Research, Nordnes, P.O. Box 1870, N-5817, Bergen, Norway

**Keywords:** Atlantic salmon, Farmed escapees, Introgression, Hybrid, Common garden, mRNA level, Insulin-like growth factor −1, Oxidative stress, Non additive inheritance

## Abstract

**Background:**

Ten generations of domestication selection has caused farmed Atlantic salmon *Salmo salar* L. to deviate from wild salmon in a range of traits. Each year hundreds of thousands of farmed salmon escape into the wild. Thus, interbreeding between farmed escapees and wild conspecifics represents a significant threat to the genetic integrity of wild salmon populations. In a previous study we demonstrated how domestication has inadvertently selected for reduced responsiveness to stress in farmed salmon. To complement that study, we have evaluated the expression of seven stress-related genes in head kidney of salmon of farmed, hybrid and wild origin exposed to environmentally induced stress.

**Results:**

In general, the crowding stressor used to induce environmental stress did not have a strong impact on mRNA expression levels of the seven genes, except for insulin-like growth factor-*1* (*IGF-1*) that was downregulated in the stress treatment relative to the control treatment. mRNA expression levels of glutathione reductase (*GR*), Cu/Zn superoxide dismutase (*Cu*/*Zn SOD*), Mn superoxide dismutase (*Mn SOD*), glutathione peroxidase (*GP*) and *IGF*-*1* were affected by genetic origin, thus expressed significantly different between the salmon of farmed, hybrid or wild origin. A positive relationship was detected between body size of wild salmon and mRNA expression level of the *IGF*-*1* gene, in both environments. No such relationship was observed for the hybrid or farmed salmon.

**Conclusion:**

Farmed salmon in this study displayed significantly elevated mRNA levels of the *IGF*-*1* gene relative to the wild salmon, in both treatments, while hybrids displayed a non additive pattern of inheritance. As *IGF*-*1* mRNA levels are positively correlated to growth rate, the observed positive relationship between body size and *IGF*-*1* mRNA levels detected in the wild but neither in the farmed nor the hybrid salmon, could indicate that growth selection has increased IGF-1 levels in farmed salmon to the extent that they may not be limiting growth rate.

## Background

The commercial production of Atlantic salmon *Salmo salar* L. was established in Norway in the 1970's [1], and each year hundreds of thousands of farmed salmon escape into the wild [2], possibly exceeding the number of wild salmon in the natural habitat. Farmed escapees have been documented to enter freshwater, and in some rivers, in some years, represent more than 80% of the total number of spawners [3]. As a consequence, genetic introgression between farmed and wild conspecifics has been documented in several rivers [4-10]. Hence, farmed escaped Atlantic salmon represents one of the largest threats to the genetic integrity of wild salmon populations.

Both directional and inadvertent selective breeding causes farmed salmon to deviate from wild populations in a range of traits, e.g., body size [11-13], body proportions [14], fat reserves [15], time of sexual maturation [16], survival [17], aggressiveness [14,18,19], predator awareness [20], neutral genetic markers [21,22], allele frequencies [23] and gene expressions [24-26]. By comparing the growth reaction norms of farmed, hybrid and wild salmon exposed to an environmentally induced stressor, we have recently demonstrated how domestication selection over approximately ten generations has inadvertently selected for reduced responsiveness to stress in farmed Atlantic salmon [27]. A number of genes have been associated with stress in salmonids and here we have evaluated the expression of seven commonly studied genes in Atlantic salmon, in salmon of farmed, hybrid and wild origin exposed to environmentally induced stress.

Five of the genes investigated in the present study are known to be regulated by oxidative stress [28,29]. These are the four antioxidant genes, glutathione reductase (*GR*), Cu/Zn superoxide dismutase (*Cu*/*Zn SOD*), Mn superoxide dismutase (*Mn SOD*), glutathione peroxidase (*GP*) and the heat-shock protein 70 (*HSP70*). Insulin-like growth factor-1 (*IGF*-*1*), a protein important in the regulation of most physiological processes in fish, including somatic growth and metabolism, is downregulated by starvation and nutritional stress and activates the insulin-like growth factor-1 receptor (*IGF*-*1R*) [30-33]. The reference gene, eukaryotic elongation factor 1 alpha (*EF1A*_*A*_) is involved in protein synthesis and has been thoroughly validated as a reliable reference gene in quantitative real time PCR examination of gene expressions in Atlantic salmon [34,35], as well as in a broad range of other organism, e.g., plants [36], copepods [37], fish [38] and humans [39].

A total of 29 families were mixed together in a common garden experiment, exposed to standard hatchery conditions or in addition environmentally induced stress, i.e., reduction of water level, twice a day for 30 minutes. Thus, our objectives were to determine the effect of environmentally induced stress upon regulation of the selected genes and further examine whether the process of domestication has caused alterations in the mRNA expression levels. Based upon the results from our previous growth study, documenting reduced responsiveness to stress in the farmed salmon studied here, we hypothesised that the farmed salmon would display attenuated regulations of the genes investigated in this study in comparison to their wild counterparts. Although the crowding stressor used in this study did not inflict a strong regulation in the mRNA expression level of the genes studier here, except for the *IGF*-*1* gene that was downregulated in the stress treatment, genetic origin had an impact on expression of five of the genes. Here we report of significant differences in mRNA levels of *GR*, *Cu*/*Zn SOD*, *Mn SOD*, *GP* and *IGF*-*1* between farmed, hybrid or wild Atlantic salmon. In the wild salmon a positive relationship was detected between *IGF*-*1* mRNA levels and body size, in both treatments, while no relationship was detected in the hybrid and farmed salmon where *IGF*-*1* levels were significantly elevated.

## Methods

Ten pure wild Atlantic salmon families, ten pure farmed families and nine F_1_ hybrid families were generated for this experiment in November 2009. Farmed parental salmon originated from the Norwegian Mowi strain, while wild parental salmon were caught by rod in the river Etne (59°40´N, 5°56´E). Hybrids were created by crossing farmed females with wild males. All families were created in the hatchery, located on the river Etne. Fertilized eggs (50 eggs/family) were mixed in four replicated tanks (n = 1450), and transported to Matre Research Station at the eyed-egg stage before hatching.

Two tanks were reared under standard hatchery conditions throughout the entire experiment running from June 3 - September 23–24, 2010. The two remaining tanks were subjected to a stressor, twice a day five days a week, in the same period. Stress was induced by a dramatic lowering of the water level for 30 minutes, hence the fish density increased although water circulation was maintained. Panic behaviour was observed as rapid movement within the tank. A stop watch was initiated when the water level was stabilized at the reduced level (3 cm). Water level during the stress treatments was adjusted throughout the experimental period in order to control for the increasing size of the fish during the experiment (5 cm depth at the time of termination). In all other aspects, the two treatments were given identical conditions throughout the experiment. These two treatments we hereon refer to as the control and stress treatments.

The experimental protocol (permit number 2648) was approved May 3, 2010, by the Norwegian Animal Research Authority (NARA).

### Sampling

The experiment was terminated after 16 weeks of treatment. Two weeks prior to termination (i.e., week 14), 750 individuals had been removed from each of the four tanks for phenotypic growth comparisons [27]. The treatments (i.e., stress and control) were maintained in weeks 14–16. At the time of terminal sampling in week 16, there were 700 individuals within each tank, minus mortality (124, 125, 77 and 105 individuals, from hatching throughout the experimental period, in tank 1, 2, 3 and 4 respectively). The terminal sample consisted of removing at random 75 individuals from each tank. This was conducted over a period of 2 days.

On the first day of the terminal sampling, a control treatment tank was sampled first, followed by a stress treatment tank, and vice versa the second day. All sampled individuals were euthanized with an overdose of benzocain (160 mg/L) (Benzoak® Vet, A.C.D Pharmaceuticals, Leknes, Norway) in a combination with metodmidat hydrochloride (10 mg/L) (Aquacalm® Vet, ScanVacc, Årnes, Norway), to inhibit the acute cortisol stress response [40]. The concentrated euthanizing agents were added to a mixture of water and ice (7:3), and the individuals were left in the solution for a maximum of 27 minutes. 25 individuals were sampled at once, leaving the experimental tanks subject to only three strokes by the landing net, 3 sampling periods, and 1 h from the first to the last stroke. Fork length and weight were measured, before the individuals were caudal fin clipped and head kidney was sampled. Fins were placed on 95% ethanol, and samples for qPCR analyses were preserved on RNAlater™. To allow the RNAlater™ to protrude into the biological tissue, the samples were stored at < 4°C for 24 h, before being transferred to −20°C.

### Microsatellite genotyping and parentage testing

280 of the individuals as sampled above were assigned to family using DNA microsatellite markers (70 individuals randomly selected per tank). Following procedures recommended by the manufacturer, DNA was extracted in 96 well plates using a Qiagen DNeasy®96 Blood & Tissue Kit. To ensure correct genotyping, parental DNA was extracted twice. On each 96-well plate, two randomly assigned blank wells were included, thus to ensure a unique identification of the plate. Six microsatellite loci were amplified in one multiplex PCR; *SsaF43* [GenBank: U37494] [41], *Ssa197* [GenBank: U43694.1] [42], *SSsp3016* [GenBank: AY372820], *MHCI*[43], *MHCII*[44] and *SsOSL85* [GenBank: Z48596.1] [45]. PCR products were run on an ABI Applied Biosystems ABI 3730 Genetic Analyser and sized-called according to the 500LIZ™ standard. Genotypes were identified using GeneMapper V4.0., with manual control of scored alleles. Assignment to family were performed by FAP Family Assignment Program v3.6 [46], using an exclusion-based approach to unambiguously identify parental origin. This program has successfully been used on several occasions for parentage testing common garden studies using these facilities [47,48]. The genetic markers analysed here have revealed very low genotyping errors in this laboratory [49] and are routinely used in association with a genotyping service for the Norwegian legal authorities to identify the farm of origin for escapees [50,51].

After DNA identification, 15 farmed, hybrid and wild individuals, respectively, within each tank were selected for the gene expression profiling. Individuals were selected by family, representing all 29 families if possible and in an even number (range 0 – 3 fish per family per tank). Choice of individuals within families on which to conduct qPCR was first based upon sampling period, then upon time in the euthanizing solution. Individuals from sampling period 1 were preferred over individuals from the subsequent periods, and within each sampling period individuals with the fewest minutes in the euthanizing solution were selected first (range 1–27 minutes). The 100 excess individuals were excluded from any further studies, thus leaving the total data set consisting of 180 individuals (45 individuals per tank).

### RNA extraction

Total RNA was extracted *in situ* from the macrodissected head kidney samples. The 180 selected individuals were randomized into 15 batches and isolated over a period of 3 days. Up to 50 mg tissue was homogenized in 1 mL TRIzol using a FastPrep homogenisator (Thermo Electron) and Lysing Matrix D ceramic beads (MP Biomedical). Following homogenization, 400 μL chloroform was added and the sample vortexed for 1 min, phase separated by centrifuge and the aqueous phase were collected using iPrep™ Purification Instrument (Invitrogen) with the iPrep™ TRIzol ® Plus RNA Kits, according to the manufacturers protocol. The RNA was eluted in 50 μL. Quantity of the isolated RNA was assessed by Nanodrop® spectrophometer (NanoDrop Technologies, Wilmington, DE). 260/280 absorbance ratio ranged from 1.61 – 2.16 with a mean average value of 2.03, while the 260/230 absorbance ratio ranged from 1.86 – 2.46 with a mean average value of 2.29. From each isolation batch minimum three samples were randomly selected, 48 samples in total, and the RNA integrity was evaluated by Agilent 2100 Bioanalyzer (Agilent Technologies, Palo Alto, CA), using RNA 6000 Nano LabChip® (Agilent Technologies, Palo Alto, CA). With a mean RNA integrity number (RIN) of 9.5 (range 8.0 – 10.0), no samples showed any sign of RNA degradation. Total RNA samples were randomized again before normalized with distilled water (dH_2_O) to a final concentration of 100 ng/μL and stored in 2 x 96 Well Plates at - 80°C.

### cDNA synthesis

For each sample cDNA synthesis were carried out in triplicate from 200 ng total RNA in 10 μL reaction volume using qScript™ cDNA Synthesis Kit (Quanta, Biosciences) in accordance to suppliers protocols. cDNA was subsequent diluted 1:10 in dH_2_O and stored at - 20°C.

Twenty-eight samples were distributed in triplicate on each cDNA 96 Well Plate. Thus, 16 samples were included a second time on one of the plates to secure full plates at all times. Negative Reverse Transcriptase Controls (nRT, a minus enzyme control**)** to control for genomic DNA contamination, was made from 200 ng total RNA from the first and the last sample on each RNA tray, and from two random samples in the middle of the RNA tray. nRTs were made from the qScript™ cDNA Synthesis Kit (Quanta, Biosciences) and contained all the reaction components except the reverse transcriptase enzyme, and were diluted and stored in the same manner as the cDNA.

Positive control (PK) was made by a mix of total RNA from all 180 samples. For this purpose 100 ng RNA per sample, in 5 μL reaction volume, were converted into cDNA in the same manner as described above, then all samples were mixed together as one PK. The Positive Control was diluted 1:20 in dH_2_O and stored at - 20°C.

### Genes and primers

Quantitative PCR primers and probes for the genes to be analyzed were obtained from published literature of earlier gene expression studies in Atlantic salmon [34,52-55]. The chosen target genes were heat shock protein 70 *HSP70* [GenBank: BG933934] [52], glutathione reductase *GR* [GenBank: BG934480] [52], Cu/Zn superoxide dismutase *Cu*/*Zn SOD* [GenBank: BG936553] [55], Mn superoxide dismutase *Mn SOD* [GenBank: DY718412] [53], glutathione peroxidase GP [GenBank: BG934453] [55], insulin-like growth factor-1 *IGF*-*1* [GenBank: M81904][54] and the insulin-like growth factor-1 receptor *IGF*-*1R* [GenBank: AY049954] [54]. Normalization of target genes was performed against the reference gene eukaryotic elongation factor 1 alpha, *EF1A*_*A*_ [GenBank: AF321836] [34]. This gene has been documented to be one of the most reliable reference genes in Atlantic salmon [34,35] and is often used as the sole reference gene in qPCR examination of gene expressions in this species. In our study amount of total RNA was equalized between samples prior to cDNA synthesis, which allowed us to statistically demonstrate that this gene was stable between all three genetic origins and between treatments (see Results). The qPCR primers and hydrolysis probe sequences are presented in Table 1.

**Table 1:** Primer and probe sequences for qPCR used in the present study

**Gene**	**GenBank #**	**Primer forward 5'-3'**	**Primer reverse 5'-3'**	**Hydrolysis probe 5'-3'**	Amplicon size (bp)*	**Reference**
*EF1A*_*A*_	AF321836	CCCCTCCAGGACGTTTACAAA	CACACGGCCCACAGGTACA	6-FAM-ATCGGTGGTATTGGAAC-MGB	57	[34]
*HSP70*	BG933934	CCCCTGTCCCTGGGTATTG	CACCAGGCTGGTTGTCTGAGT	6-FAM-CGCTGGAGGTGTCATG-MGB	121	[52]
*GR*	BG934480	CCAGTGATGGCTTTTTTGAACTT	CCGGCCCCCACTATGAC	6-FAM-AGCCTTCCTAAGCGCAG-MGB	61	[52]
*Cu*/*Zn SOD*	BG936553	CCACGTCCATGCCTTTGG	TCAGCTGCTGCAGTCACGTT	6-FAM-ACAACACCAACGGCT-MGB	140	[55]
*Mn SOD*	DY718412	GTTTCTCTCCAGCCTGCTCTAAG	CCGCTCTCCTTGTCGAAGC	6-FAM-CACATCAACCACACCATCTTCTGGACAAAC-TAMRA	209	[53]
*GP*	BG934453	GATTCGTTCCAAACTTCCTGCTA	GCTCCCAGAACAGCCTGTTG	6-FAM-TGAATGGAGACACAGAAC-MGB	140	[55]
*IGF*-*1*	M81904	GTGTGCGGAGAGAGAGGCTTT	TGTGACCGCCGTGAACTG	6-FAM-TTTCAGTAAACCAACGGGCTATGG-TAMRA	68	[54]
*IGF*-*1R*	AY049954	TGAAGAGCCACCTGAGGTCACT	TCAGAGGTGGGAGGTTGAGACT	6-FAM-CGGGCTAAAGACCCGTCCCAGTCC-TAMRA	72	[54]

*bp = base pair.

### Quantitative real-time PCR (qPCR)

qPCR was performed in triplicates in 14 × 384 Well Plates on ABI 7900HT Fast Real-Time PCR System (Applied Biosystems) in 5 μL reaction volume with 1.5 μL cDNA template and Briliant III Ultra-Fast QPCR Master Mix. Primers and probes had a final concentration of 900 μM and 200 μM, respectively. A passive reference dye, ROX™, was included in the reaction mix. On each 384 Well Plate all 8 genes were run with 14 samples in triplicate. For each plate one No Template Control (NTC), two different nRTs and three PKs were included for each gene. NTCs contained all reaction components besides template (cDNA substituted by dH_2_O) and were added to monitor possible PCR contaminations and primer dimer formations. All genes had previously been validated, thus their efficiency documented to be approximately the same [34,52-55].

Quantification cycle values (Cq) were obtained from the qPCR instrument using SDS (2.4) and RQ Manager (1.2.1) (Applied Biosystems). Baseline and threshold for Cq values were set manually for each gene and kept identical for all plates. One Cq equals a doubling (2^^Cq^) of the mRNA level.

The experiment was performed in accordance to the general guidelines for qPCR experiments, Minimum Information for Publication of Quantitative Real-Time PCR Experiments “MIQE” [56,57].

### Statistical analysis

The comparative quantification cycle (Cq) method [58], were used to analyse the relative gene expression of the target genes. The median ΔCq value of the wild salmon in the control treatment was used as calibrator when calculating ΔΔCq values. ΔΔCq values were multiplied by −1, so that upregulated mRNA levels were displayed as positive values, while downregulated mRNA levels were displayed as negative values.

Quantification cycle values, Cq, were quality checked, first by manually removing non-amplified samples, samples displaying extreme Cq values (Cq <15 and > 39) and aberrant Cq values caused by documented sampling errors. Outliers defined as values more than 1.5 times the interquartile range (IQR) above the 3rd quartile and below the 1st quartile [59] were excluded from the data set. Possible outliers were identified based upon several calculated interquartile ranges; IQR of the Cq values of each target gene and the reference gene, IQR of the standard deviation (SD) of the Cq values and IQR of the Cq, ΔCq and the ΔΔCq values, of each target gene in each treatment. Samples had to pass all the selected criterions to be included in the statistical analysis. For passed samples, the median of the three replicates were used as the sample’s Cq value. For the 16 samples that were run twice, on two different plates, the mean of the two collapsed triplicates were used as the samples Cq value.

Linear mixed effect models (LME), testing for difference in continuous response variables, were used to model variation in weight at termination and mRNA expression levels between treatments and genetic origin. LMEs were fitted for Cq values of the reference gene and ΔΔCq values of the target genes. Model selection was performed based upon Akaike information criterion (AIC) values, calculated using the restricted maximum likelihood (REML) [60]. Models displaying less than 2 AIC values in distance were considered equally good. Thus, by the principle of parsimony, the simplest model that performed best was applied. The initial model fitted for weight included treatment and type as fixed effects, as well as the interaction between them. By forward selection the random effect of tank nested within treatment, as well as a genetic (co)variance matrix across treatments were incorporated if this improved the fit of the model. Due to differences in growth rate between salmon of farmed, hybrid and wild origin and to achieve normality, the response variable, weight at termination, was log transformed (log_10_) [61-63]. A similar model with treatment and type as fixed effects (and the interaction between them) were fitted for the expression of each of the eight genes (Cq and ΔΔCq values). By forward selection the random effect of tank nested within treatment, a genetic (co)variance matrix, log-weight of fish, sampling period, and minutes in anaesthesia were incorporated if this improved the fit of the model. To satisfy homogeneity and normality in the model, Cq and ΔΔCq values were log transformed. Prior to transformation, ΔΔCq values were added a constant so that all values were above 1. For AIC comparisons of LME models, see Additional file 1. Gene expression in farmed versus hybrid salmon, hybrid versus wild salmon and wild versus farmed salmon were compared by re-running the models while excluding one of the three genetic origins at a time. For the re-runs, multiple comparisons were counteracted by the Bonferroni correction, giving an adjusted significance level of P < 0.017. P-values are given from F-statistics of the simplest model. Numerator degrees of freedom were given as k – 1, where k is the number of factor levels. Denominator degrees of freedom were calculated as N – k, where N was set to the smallest sample size detected in any of the three genetic origins in any of the two treatments. Linear regressions between ΔΔCq values on the y-axis, and weight of fish (g), sampling period (1–3) and time in anaesthesia (minutes) on the x-axis, were performed with a 95% confidence interval. The goodness of fit of the linear regression was validated by the R-square values and by the P-values of the slopes. As we measured 7 genes, multiple comparisons was counteracted by the Bonferroni correction, which in this case gave an adjusted P-value of 0.007.

All statistical analysis was performed using R ver. 2.15.2 [64] with critical *P*-values set to 0.05, unless otherwise stated. LME’s were fitted using the *lmer* function in the lme4 package [65].

## Results

### Growth of experimental fish

**Figure 1:**
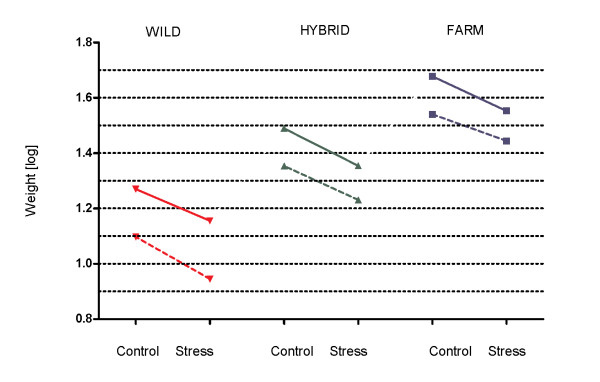
**Growth reaction norms of salmon of farmed, hybrid and wild origin.** Reaction norms for the log transformed weight measurements of Atlantic salmon of wild , hybrid and farmed origin reared in the control treatment and the stress treatment at week 14 (dotted line) of the experimental period and at termination, week 16 (solid line). Replicated tanks are pooled. The within-tank biomasses were dramatically reduced at week 14, thus the dotted (−−) and the solid (^__^) lines display the reaction norm before and after the reduction, respectively.

The mean weight, length and condition factor of farmed, hybrid and wild salmon in all four tanks is shown in Table 2. Salmon in the control treatment were significantly larger than salmon in the stress treatment and farmed salmon were significantly larger than the hybrid and wild salmon, in both treatments (Table 3; Additional file 2). At the time of sampling the effect of the stress treatment was similar in all groups, as they displayed similar growth reaction norm slopes (Figure 1, solid lines; Table 2), thus the interaction between treatment and type were not included in the final LME model (Table 3).

**Table 2:** **Growth measurements of *Salmo salar *L. of wild, hybrid and farmed origin**

**Group**	**Treatment**	**Tank**	**Measurements at termination (week 16)**										**Weight difference**	
			**n**	**Mean W (g)**	**± SD**	**Mean L (cm)**	**± SD**	**Mean K**	**± SD**	**n**	**Mean W (g)**	**± SD**	**Absolute (g)**	**Percent (%)**
**Wild**														
	Control	1	15	21.07	9.58	11.63	2.08	1.28	0.05	30	18.6	8.8	4.3	23.12
		2	15	16.13	7.44	10.71	1.76	1.27	0.07					
	Stre**s**s	3	15	14.47	6.77	10.34	1.74	1.24	0.05	30	14.3	6.48		
		4	15	14.13	6.4	10.42	1.63	1.18	0.05					
**Hybrid**														
	Control	1	15	30.73	10.67	13.29	1.49	1.32	0.1	30	30.83	9.81	8.23	26.69
		2	15	30.93	9.24	13.65	0.6	1.24	0.32					
	Stress	3	15	22.8	5.82	12.23	1.15	1.23	0.06	30	22.6	5.97		
		4	15	22.4	6.35	12.13	1.23	1.24	0.04					
**Farm**														
	Control	1	15	46.47	8.21	15.22	0.9	1.31	0.04	30	47.57	9.58	11.9	25.02
		2	15	48.67	10.97	15.47	1.18	1.29	0.06					
	Stress	3	15	33.67	3.96	13.93	0.5	1.28	0.05	30	35.67	5.74		
		4	15	37.67	6.64	14.32	0.82	1.29	0.04					

Weight (gram), length (cm) and condition factor (K) with standard deviations. Differences in weight between treatments; Absolute (grams); Percent (percent reduction in weight in the stressed environment, compared to in the control environment).

**Table 3:** The effect of treatment, type and their interaction on fish weight and gene expressions

***Salmo salar *L.**	**n**	**Effects**						
		**Fixed**	**Random**	**DFn**	**DFd**	**Sum Sq**	**F**	**P**
Weight [log]	180	Treatment	Treatment:Tank	1	28	0.62	23.19	<0.0001***
		Type		2	27	6.06	113.11	<0.0001***
Gene	n			DFn	DFd		F	P
*EF1A A*	177	Type	Treatment:Tank	2	26	0.0002	1.17	0.3
*HSP70*	173	Treatment	Treatment:Tank	1	26	0.0017	0.72	0.3
*GR*	173	Type	Treatment:Tank	2	25	0.08	7.92	0.001***
*Cu*/*Zn SOD*	166	Type	Treatment:Tank	2	24	0.06	3.49	0.04*
*Mn SOD*	170	Type	Weight [log]	2	24	0.03	5.42	0.01**
*GP*	172	Type	Weight [log]	2	24	0.07	6.79	0.003**
*IGF*-*1*	168	Treatment	Weight [log]	1	25	0.09	13.57	<0.001***
		Type		2	24	0.15	10.97	<0.001***
*IGF*-*1R*	173	Type	Treatment:Tank	2	23	0.01	1.37	0.24

Summary of the best linear mixed effect models testing for difference in fish weight, and mRNA levels (raw Cq values of EF1A_A,_ ΔΔCq values of target genes) between treatments and genetic origins (type). Including the interaction between treatment and type did not improve the fit of any of the models, thus all groups were affected by treatment in the same matter. The random effects were included by forward selection. Treatment:Tank; tank nested within treatment, Weight [log]; log transformed weight measurement of the fish. For more information on model selection based upon AIC values, see Additional file 1. The statistical significance is marked with asterisks, where * = P ≤ 0.05, ** = P ≤ 0.01 and *** = P ≤ 0.001.

### Quality of qPCR

The expression of the reference gene *EF1A*_*A*_ (raw Cq values) was stable between farmed, hybrid and wild salmon in both treatments (Table 3). The average mean Cq value of samples before and after quality check, displayed a minor deviation of 0.02, while the median was identical (Table 4). Collapsing samples where all triplicates passed the selected quality criterions resulted in 98% of the reference gene samples and 95% of the target gene samples being included in the statistical analysis.

**Table 4:** **Variation of raw EF1A**_**A **_**Cq values**

**Quality checked**	**n**	**Median**	**± SD**	**Mean**	**Min**	**1st Qu**	**3rd Qu**	**Max**	**NA**
No	576	22.13	0.53	22.19	20.90	21.85	22.48	26.16	12*
Yes	177	22.13	0.47	22.17	21.04	21.89	22.43	23.92	3

* 6 runs not amplified (2 individuals), 6 runs removed due to a sampling error (1 individual). Triplicates collapsed after quality check. 1^st^ Qu; lower quartile (25 percentile), 3^rd^ Qu; upper quartile (75 percentile).

For gene *HSP 70*, 64% of the negative Reverse Transcriptase Controls (nRTs) turned out positive due to amplification of genomic DNA. The HSP70 assay is based upon an EST sequence of the gene, hence no information on the exon-exon junctions was used in assay design, which likely explains these results. However, this did not impose a problem in analysing the data, as the difference between the average Cq value for positive nRTs and the samples average Cq value were larger than 9. Ignoring *HSP70*, nRTs were negative in 98% of the cases, and the difference between average Cq value for positive nRTs and the samples average Cq value were larger than 10 in all positive nRTs (10, 10, 10 and 12, respectively). Positive controls PK’s were amplified in > 99% of the controls, while No Template Controls NTC’s turned out non-amplified in > 97% of the controls. The NTCs that were amplified displayed a Cq value of +10, + 13 and +14, when compared to the adjoining samples average Cq value.

### The effect of treatment and tank

**Figure 2:**
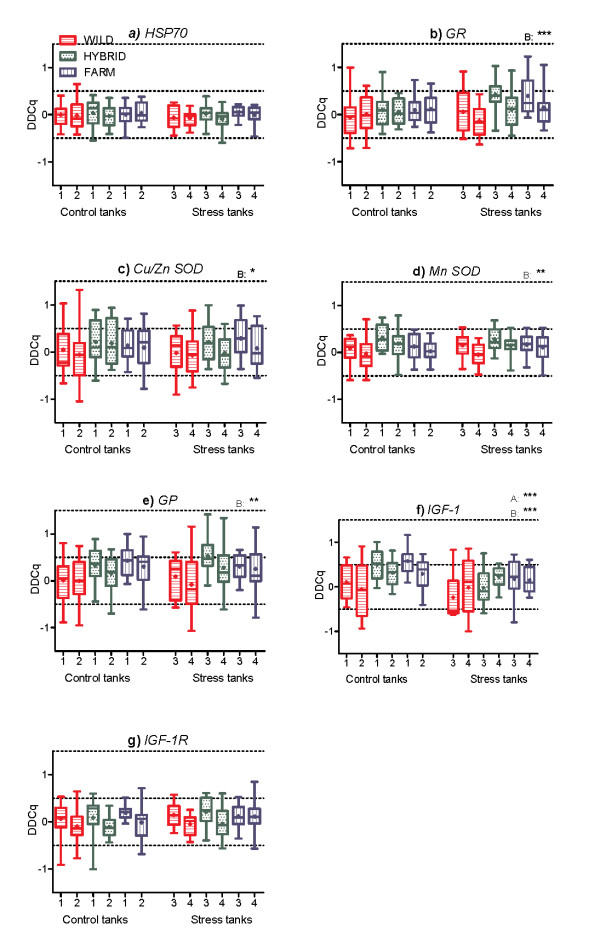
**qPCR analyses of the seven selected genes in salmon of farmed, hybrid and wild origin.** Expressions of **a**) *HSP70*, **b**) *GR*, **c**) *Cu*/*Zn SOD*, **d**) *Mn SOD*, **e**) *GP*, **f**) *IGF*-*1* and **g**) *IGF*-*1R* in Atlantic salmon of wild, hybrid and farmed origin, reared in a standard hatchery environment (tank 1 and 2) or in addition exposed to environmentally induced stress (tank 3 and 4). One quantification cycle (Cq) equals a doubling of the amount of mRNA (2^^Cq^). All values are relative to the wild salmon in control treatment (pooled), and ΔΔCq values with positive and negative values indicate upregulated and downregulated mRNA levels, respectively. Boxes show the median (thick line), mean (+), 1^st^ and 3^rd^ quartiles (lower and upper boundary) and the lower and upper extreme (whiskers). The statistical significance of the effect of treatment (A) and type (B) on gene expressions is marked with asterisks, where * = P ≤ 0.05, ** = P ≤ 0.01 and *** = P ≤ 0.001.

Some tank effects were detected (Figure 2a-c, g). Statistically, this was controlled for by including the random effect of tank nested within treatment in the linear mixed effect models, which significantly improved the fit of the models (Table 3). With the exception of the *IGF*-*1* gene, that was downregulated in the stress treatment, the genes investigated in this study were not upregulated, nor downregulated by the environmentally induced stress (Table 3). Thus, treatment, i.e., control/stress, did not have a significant effect upon mRNA expression levels, except for the *IGF*-*1* gene where the median ΔΔCq value displayed in the control treatment were downregulated by −0.23 in the stress treatment (Figure 2f; Additional file 3).

### The effect of genetic origin (farm/hybrid/wild)

In five of the genes, *GR*, *Cu*/*Zn SOD*, *Mn SOD*, *GP* and *IGF*-*1*, mRNA expression levels were significantly different between the genetic origins (Table 3, Figure 2b-f). Thus, mRNA expression levels of *HSP70* and *IGF*-*1R* were not significantly different between salmon of farmed, hybrid and wild origin (Table 3, Figure 2a, g).

Farmed salmon displayed elevated mRNA expression levels of *GR*, *CuZn SOD*, *GP* and *IGF*-*1*, relative to the wild salmon (Table 5, Additional files 2 and 3). *Mn SOD* was expressed insignificantly different in the farmed and wild salmon (Table 5, Additional files 2 and 3).

**Table 5:** Median ΔΔCq values of the seven genes in farmed and hybrid-, relative to wild salmon

**Group**	***HSP70***		GR***		*Cu*/ ZnSOD *		MnSOD**		GP**		*IGF*- 1 ***		***IGF*-*1R***	
Wild	0	(a,a)	0	(a,a)	0	(a,a)	0	(a,a)	0	(a,a)	0	(a,a)	0	(a,a)
Hybrid	0.01	(a,a)	0.27	(b,b)	0.13	(b,ab)	0.17	(b,b)	0.32	(b,b)	0.32	(b,b)	0.00	(a,a)
Farm	0.03	(a,a)	0.18.	(b,b)	0.15	(b,b)	0.09	(a,a)	0.35	(b,b)	0.36	(b,b)	0.07	(a,a)

Upregulated mRNA levels are displayed as positive values (multiplied by −1). The statistical significance of the effect of genetic origin on gene expressions, in general, is marked with asterisks, where * = P ≤ 0.05, ** = P ≤ 0.01 and *** = P ≤ 0.001. Letters in brackets represent statistical significance among the three origins, the first with the significance level set at P ≤ 0.05 and the second with the Bonferroni correction for multiple comparisons, P ≤ 0.017.

For the hybrid salmon, mRNA levels of the *GR*, *Cu*/*Zn SOD*, *GP* and *IGF*- *1* gene were similar to the mRNA levels detected in the farmed salmon (Table 5, Additional files 2 and 3). When the significance levels were adjusted for multiple comparisons, *Cu*/*Zn SOD* was in addition expressed insignificantly different between hybrid and wild salmon, P = 0.023 (Table 5, Additional files 2 and 3). For *Mn SOD*, hybrids displayed elevated mRNA levels, in comparison to both farmed and wild salmon (Table 5, Additional files 2 and 3). Thus, hybrids displayed three out of five genes similar to farmed salmon, one gene intermediate to farmed and wild salmon, and one gene significantly elevated compared to both farmed and wild salmon (Table 5).

### The influence of fish size and sampling

The relationship between mRNA expression levels and fish size, sampling period, and time in anesthesia, for all three groups in both treatments, is shown in Table 6. The influence of fish size was significant for four genes in the control treatment, and for three genes in the stress treatment. Interestingly, these trends were only observed in the wild fish, and all relationships between gene expression and fish size were positive (Table 6; Additional file 4).

**Table 6:** Linear regression between gene expression and fish weight, sampling period and time in anaesthesia

	***HSP70***		***GR***		***Cu*/*Zn SOD***		***Mn SOD***		**GP**		**IGF-1**		**IGF-1R**	
	**R square**	**P**	**R square**	**P**	**R square**	**P**	**R square**	**P**	**R square**	**P**	**R square**	**P**	**R square**	**P**
Weight														
Control														
Wild	0.046	0.265	0.046	0.254	0.250	0.005 **	0.290	0.002 **	0.154	0.036 *	0.320	0.002 **	0.000	0.917
Hybrid	0.018	0.492	0.028	0.393	0.011	0.601	0.023	0.440	0.014	0.547	0.038	0.311	0.037	0.306
Farm	0.002	0.798	0.066	0.178	0.021	0.463	0.015	0.533	0.007	0.663	0.023	0.442	0.062	0.201
Stress														
Wild	0.095	0.111	0.161	0.034 *	0.002	0.809	0.004	0.746	0.190	0.023 *	0.324	0.001 **	0.012	0.602
Hybrid	0.060	0.192	0.029	0.379	0.017	0.519	0.018	0.500	0.090	0.108	0.034	0.360	0.058	0.208
Farm	0.007	0.671	0.002	0.821	0.028	0.405	0.027	0.407	0.001	0.898	0.000	0.986	0.010	0.607
Period														
Control														
Wild	0.015	0.524	0.039	0.293	0.022	0.438	0.002	0.836	0.049	0.250	0.005	0.731	0.010	0.607
Hybrid	0.090	0.120	0.018	0.498	0.031	0.383	0.002	0.814	0.022	0.448	0.067	0.176	0.022	0.439
Farm	0.178	0.023 *	0.021	0.455	0.007	0.678	0.033	0.343	0.037	0.325	0.053	0.238	0.070	0.173
Stress														
Wild	0.033	0.356	0.019	0.488	0.020	0.483	0.000	0.951	0.053	0.249	0.047	0.257	0.000	0.993
Hybrid	0.057	0.204	0.058	0.209	0.001	0.862	0.000	0.961	0.001	0.847	0.036	0.346	0.139	0.047*
Farm	0.070	0.165	0.006	0.678	0.054	0.244	0.023	0.439	0.009	0.634	0.028	0.407	0.012	0.566
Minutes														
Control														
Wild	0.217	0.011 *	0.011	0.587	0.051	0.231	0.083	0.123	0.113	0.074	0.050	0.252	0.130	0.050 *
Hybrid	0.001	0.859	0.006	0.695	0.002	0.836	0.010	0.614	0.032	0.354	0.008	0.643	0.005	0.721
Farm	0.007	0.666	0.002	0.806	0.007	0.668	0.056	0.215	0.005	0.714	0.158	0.036 *	0.030	0.382
Stress														
Wild	0.008	0.643	0.025	0.424	0.006	0.706	0.002	0.816	0.014		0.001	0.891	0.023	0.462
Hybrid	0.006	0.682	0.008	0.650	0.002	0.830	0.050	0.252	0.026	0.395	0.013	0.570	0.079	0.140
Farm	0.000	0.979	0.012	0.570	0.099	0.110	0.002	0.821	0.002	0.819	0.070	0.183	0.001	0.890

* P < 0.05; ** P < 0.007; Weight (gram); Period (1–3); Time (1–27 minutes).

A significant positive relationship was detected between mRNA expression levels and sampling period in two of the genes, although only for one group, in one treatment (Table 6; Additional file 5). The effect of time in anaesthesia was significant in three genes, although for all genes the negative relationship between gene expression and sampling period was only displayed in one group, and only in one of the treatments (Table 6; Additional file 6).

**Figure 3:**
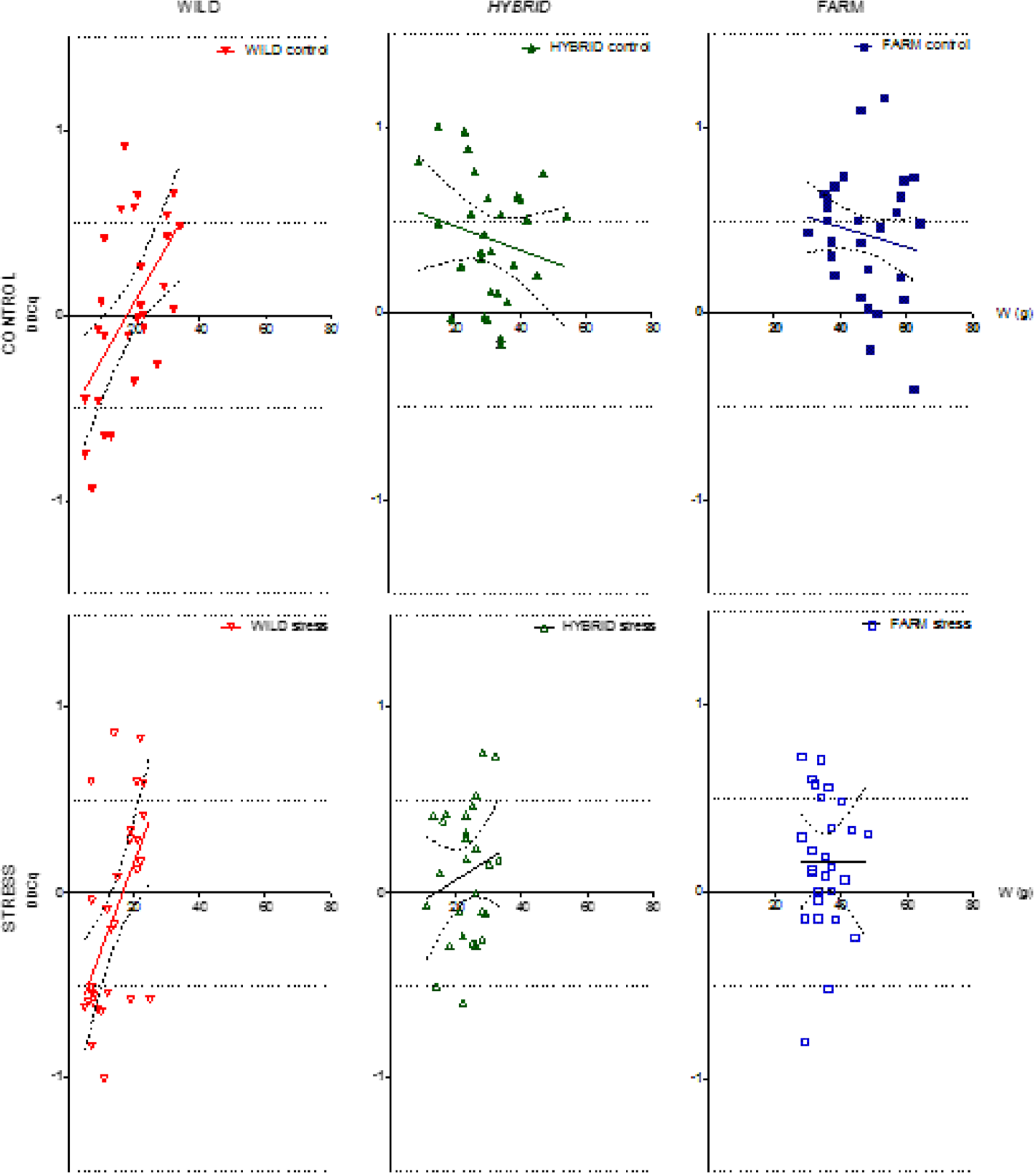
**Linear regressions between expression of the *IGF*-*1 *gene and fish weight in Atlantic salmon.** ΔΔCq value of the *IGF*-*1* gene plotted against fish weight, for salmon of wild, hybrid and farmed origin, in the control treatment and the stress treatment, replicated tanks are pooled. A significant positive relationship was detected in the wild salmon, in both treatments. The regression line is shown with a 95% confidence interval.

Overall, 9 out of the 12 significant regressions detected here, and all of the regressions detected between mRNA level and fish weight, were displayed in the wild salmon. When adjusted for multiple testing, the adjusted P-values displayed only significant relationships between the expression of three genes and fish size in the wild salmon. For *Cu*/*Zn SOD* and *Mn SOD* the significant positive relationship were only detected in the control treatment. However, the relationship between fish size and *IGF*-*1* was significant for wild salmon in both treatments (r^2^ = 0.320 and 0.324, in the control and stress treatment, respectively) and as the significant regression between gene expression and fish size was positive, the largest wild salmon displayed the highest mRNA expression levels of the insulin-like growth factor-I *IGF*-*1*gene (Figure 3).

## Discussion

Overall, no significant differences in mRNA expression of the seven genes investigated here were detected in salmon reared under standard hatchery conditions and salmon exposed to environmentally induced stress. One exception was detected in the insulin-like growth factor-1 (*IGF*-*1*), which was significantly different between treatments. As the mRNA expression level of the *IGF*-*1* gene was downregulated in the stress treatment relative to the control treatment, as well as growth being lower, this indicates that nutritional stress [31,32,66-69], e.g., impaired feed intake, was induced in this study. The corresponding receptor *IGF*-*1R* was however, similarly expressed in both treatments. Expression of the four antioxidant genes, glutathione reductase (*GR*), Cu/Zn superoxide dismutase (*Cu*/*Zn SOD*), Mn superoxide dismutase (*Mn SOD*), glutathione peroxidase (*GP*), as well as the heat-shock protein 70 (*HSP70*) were similar among treatments, thus oxidative stress was not detected [28,29]. In general, the crowding stressor used to induce environmental stress upon salmon in this study had no clear effect upon mRNA expression levels of the genes studied here, and we were therefore not able to verify our hypothesis by evaluating the selected genes. However, despite little to no significant differences in mRNA expression levels between treatments there could still be regulations at the protein level. Further, as Atlantic salmon is partially tetraploid [70], differences in mRNA levels between treatments could potentially be masked if gene copies are regulated differently.

Significant differences in mRNA levels between farmed, hybrid and wild salmon were detected in the antioxidant genes *GR*, *Cu*/*Zn SOD*, *Mn SOD*, and *GP*, as well as in *IGF*-*1*. Thus, genetic origin of the salmon used in this study had an impact on the mRNA expression levels of five of the seven genes investigated. Here we discuss the genes where mRNA levels were affected by genetic origin, with a primary focus on the *IGF*-*1* gene. *IGF*-*1*, in addition to being expressed significantly different between treatments and origins, also displayed a positive relationship to fish size in the wild salmon, in both treatments, while no such relationship were detected in the farmed and hybrid salmon where *IGF*-*1* levels were significantly elevated.

### Insulin- like growth factor −1

In salmonids, as well as in other fish species, positive correlations between feed ration and IGF-1 plasma levels [32,66-69,71], as well as *IGF*-*1* mRNA levels in liver [33] and muscle [72,73], has been documented. As salmonids have been documented to reduce their feeding activity as a response to stress [14,19,74], the *IGF*-*1* mRNA levels were expected to be downregulated in the stress treatment. In accordance to this expectation, the *IGF*-*1* mRNA levels were downregulated in the stress contra the control treatment in this study. This indicates that feeding activity was suppressed in the salmon exposed to environmentally induced stress.

Head kidney *IGF*-*1* mRNA levels were significantly elevated in the farmed and hybrid salmon relative to the wild salmon studied here, in both treatments, which was expected as there is documented a positive relationship between *IGF*-*1* mRNA levels, as well as plasma levels, and growth rate in salmonids and other teleosts [54,66,69,71,75-77]. This indicates that growth-selection for approximately ten generations has not only resulted in increased growth rates in farmed salmon, but also elevated *IGF*-*1* mRNA levels. Consistent with our results, elevated *IGF*-*1* mRNA levels have been observed in domesticated relative to wild coho salmon *Oncorhynchus kisutch*, in both liver and muscle tissue [78,79]. Plasma IGF-1 levels were also elevated in the domesticated relative to the wild coho salmon [79], which also has been documented in other salmonids, i.e., rainbow trout *Oncorhynchus mykiss*[80]. In contrast to these studies, no differences in plasma IGF-1 levels [81,82], nor *IGF*-*1* mRNA levels in liver, muscle or gill [81] of farmed and wild Atlantic salmon has been detected. The difference in the results between the study conducted by Neregard and colleagues [81] and the present study could be caused by tissue specific differences in regulation of *IGF*-*1* mRNA levels [83,84]. However, in their study [81], mRNA levels, as well as plasma IGF-1 levels, were measured in Atlantic salmon sampled at cold temperatures < 5°C. As plasma IGF-1 levels have been documented to decline with decreasing temperatures [67,75,85], the relatively low plasma IGF-1 levels detected in their study could make variations among the strains harder to detect. This may also explain the differences between their study and other studies documenting differing *IGF*-*1* mRNA levels in domesticated and wild salmonids.

Plasma IGF-1 levels have been documented to be correlated with body size [80]. However, the relationship between IGF-1 and body size seems to be weaker than the relationship between IGF-1 and growth rate [69,75,85,86]. Thus, IGF-1 is an indicator of growth performance at the time of measuring. A clear and positive relationship between *IGF*-*1* mRNA expression level and body size was detected in the wild salmon in both environments in this study. In contrast, no such relationship was observed for either the farmed or the hybrid salmon. Theoretically, this striking contrast could have been caused by sudden differences in growth rates between small and large wild fish at the time of sampling. Alternatively, this may reflect genetic differences between wild and farmed salmon in the way in which IGF-1 influences growth rate. First, the potential for genetic differences influencing this trend are discussed.

As farmed salmon display higher mRNA levels of *IGF*-*1* than wild fish, it appears that selection for growth has increased the growth hormone GH:IGF-1 pathway activity which is the main endocrine regulator of growth in salmonids [30]. In turn, this could explain the lack of relationship between fish size and *IGF*-*1* mRNA levels in the farmed salmon studied here as these elevated levels may not be limiting growth rate. In support of this suggestion is the fact that domesticated Atlantic salmon display a smaller growth-response to GH treatment than wild salmon [81]. This theory is further supported by the study of Devlin and colleagues [79], where mRNA levels of *IGF*-*1*, as well as other genes involved in growth regulation, was regulated alike in farmed and GH transgenic coho salmon.

In addition to potential genetic differences causing the clear difference in relationship between fish size and *IGF*-*1* mRNA levels between farmed and wild salmon, it is possible that this difference may have been caused by specific conditions in the present experiment. Farmed salmon outgrew wild salmon by 2.56:1 in the control treatment, and 2.49:1 in the stress treatment, while hybrids were outgrown by 1.66:1 and 1.58:1, respectively. Two weeks earlier the corresponding numbers were 2.93:1 and 3.42:1 for the wild salmon, and 1.54:1 and 1.61:1 for the hybrid salmon (based upon more than 2000 individuals sampled for a comprehensive growth reaction norm study, [27]. Thus, the difference in weight between wild and farmed salmon decreased after the first samples were taken, while the weight difference between hybrid and farmed salmon were stable. Also, similar growth reaction norm slopes were detected in salmon of all origin in this study, in contrast to the salmon sampled two weeks earlier, where the wild salmon displayed a significantly steeper slope than the farmed salmon (Figure 1). This could indicate that wild salmon in addition to displaying a positive relationship between *IGF*-*1* levels and body size, displayed an increased growth rate at the time of sampling. This could be due to biased sampling, if the smallest individuals were unintentionally left in the tank at the time of sampling and therefore not used in the study. Furthermore, a sudden increase in growth rate could be caused by compensatory growth, where accelerated growth rates are experienced after a period of growth depressions [87,88]. In this study, salmon of all origin were communally reared in order to avoid strain-specific tank effects. As farmed salmon are more competitive and aggressive than wild salmon [14,18,19], growth depression could unintentionally have been induced in the wild salmon in this study, due to high inter-strain competition for feed. As the within-tank biomass was significantly reduced two weeks prior to our terminal sampling, this might have caused a reduction in the competition level, causing a sudden increase in feeding activity in the wild salmon. Increased *IGF*-*1* mRNA levels in muscle of starved salmonids have previously been documented as a response to re-feeding [72,73]. Thus, compensatory growth could also, in part, explain the positive relationship between *IGF*-*1* mRNA levels and body size detected in the wild salmon.

### Antioxidant genes

In general, oxidative stress was not detected in the salmon exposed to the crowding stressor in the present study. Although mRNA expression levels were not upregulated or downregulated as a response to treatment, the four antioxidant genes, *GR*, *Cu*/*Zn SOD*, *Mn SOD* and *GP*, were expressed significantly different between the origins. In fish, oxidative stress can be induced by abiotic factors like toxins in the water [26], dissolved oxygen [53,89,90], temperature [38] and diet type [52], as well as biotic factors like age and feeding behaviour [91]. In the present study, water circulation was maintained during stressing, thus avoiding alterations in dissolved oxygen levels. However, oxidative stress generated by starvation or food deprivation has been documented in fish [92-94]. Thus, if food deprivation/compensatory growth were unintentionally induced in this study, this could have had an impact of the mRNA expression levels of the antioxidant genes that were expressed significantly different between the origins. However, differing antioxidant defense responses to starvation has been documented in salmonids when studied with respect to enzymatic activity of GR, SOD and GP [92,93,95]. For instance, a decrease in liver GR, SOD and GP enzymatic activity were detected in starved rainbow trout [92,95], while in contrast enzymatic activity in liver of brown trout increased during starvation [93], thus making it hard to generalize on the effect of oxidative stress, induced by food deprivations, in salmonids.

In this study, mRNA expression level of the antioxidant stress gene *Mn SOD* was expressed similar in the farmed and wild farmed salmon, while *GR*, *Cu*/*Zn SOD* and *GP*, were significantly elevated in the farmed relative to the wild salmon. This result is in contrast to a common-garden study documenting *GR* mRNA expression levels in Atlantic salmon originating from a domesticated Canadian strain, a wild Canadian strain and their first generation hybrids, reared under standard hatchery conditions [26]. In the study by Debes and colleagues [26] wild salmon displayed elevated mRNA levels compared to the farmed and hybrid salmon. Although, consistent with our study, hybrid and domesticated salmon displayed similar *GR* mRNA levels [26].

### Hybrids

Hybrid salmon displayed body weights at an intermediate level of the wild and farmed salmon, however the mRNA levels expressed in head kidney tissue were only displayed at an intermediate level in one of the five genes regulated in this study. In three of the genes, mRNA expression levels were similar to the levels observed in the farmed salmon, while in one of the genes, expression levels were elevated compared to both the farmed and the wild salmon.

Non additive gene expression profiles in hybrids has been documented in hybrids created from wild and farmed Atlantic salmon strains of Norwegian [24] and Canadian [96] origin. Based on these studies [24,96] and on studies on other organisms, e.g., *Drosophila*[97], maize [98], Pacific oysters *Crassostrea gigas*[99], it has been suggested that most gene expression profiles appears to be regulated as non additive traits, while most phenotypic traits, e.g., growth, display additive genetic variation. However, in contrast, other studies have documented larger portions of additive relative to non additive pattern of inheritance of gene expression profiles in both Atlantic salmon [26] and maize [100], as well as in mice [101].

When quantified by microarrays in liver tissue [96] and whole fry [24], more than 80% of genes regulated in farmed, relative to wild Atlantic salmon displayed gene expressions in hybrids that departed from additive inheritance. However, in a microarray performed on gill tissue of Atlantic salmon, only one third of the expressions regulated in gill of farmed relative to wild salmon displayed a non additive pattern of inheritance in the hybrids [26]. In the study by Normandeau and colleagues [96], as well as in the present study, non additive expression levels in hybrids were similar to the expressions of farmed salmon, while in the study by Debes and colleagues [26], the majority of the non additive expression levels were displayed at levels closer to the wild than the farmed salmon. The presence of both additive and non additive gene regulations in hybrids, as well as non additive expressions being displayed similar to both farmed and wild origin, suggest that the pattern of inheritance in gene expression profiles in Atlantic salmon is both gene and tissue-specific [26].

## Conclusions

In general, mRNA expression levels of the seven selected genes investigated in this study were not differentially regulated between treatments. One exception was detected in the *IGF*-*1* gene, which was downregulated in the stress treatment where growth was lower. Although the effect of treatment was weak, genetic origin had an effect upon mRNA expression levels of the four antioxidant genes *GR*, *Cu*/*Zn SOD*, *Mn SOD*, and *GP*, as well as *IGF*-*1*. The farmed Mowi strain displayed elevated mRNA levels for *GR*, *Cu*/*Zn SOD*, *GP*, and *IGF*-*1*, compared to the wild Etne strain, while *Mn SOD* was expressed at a similar level. Hybrids displayed both additive and non additive gene regulations.

In the wild salmon, a clear positive relationship between *IGF*-*1* mRNA expression levels and body size was observed in both replicates in both treatments. This is in contrast to the farmed and hybrid salmon where no such relationship was detected. It is not possible to exclude the possibility that this was caused by large wild salmon displaying increased growth rates at the time of sampling. However, it is suggested that the most plausible explanation for this clear difference is that as farmed salmon display higher levels of *IGF*-*1* than the wild fish, these elevated levels may not be limiting growth rate. This deserves further scientific attention.

### Availability of supporting data

The data set supporting the results of this article are available as an additional file (Additional file 7).

## Abbreviations

mRNA: Messenger ribonucleic acid;GR: Glutathione reductase;Cu/Zn SOD: Cu/Zn superoxide dismutase;Mn SOD: Mn superoxide dismutase;GP: Glutathione peroxidase;HSP70: Heat-shock protein 70;IGF-1: Insulin-like growth factor-I;IGF-1R: Insulin-like growth factor 1 receptor;EF1AA: Elongation factor 1 alpha _A_;NARA: Norwegian Animal Research Authority;DNA: Deoxyribonucleic acid;RIN: RNA integrity number;dH20: Distilled water;nRT: Negative reverse transcriptase;PK: Positive control;NTC: No template control;qPCR: Quantitative real time polymerase chain reaction;MIQE: Minimum Information for Publication of Quantitative Real-Time PCR Experiments;Cq: Quantification Cycle;IQR: Interquartile range;SD: Standard deviation;LME: Linear mixed effect models;AIC: Akaike information criterion;RMLE: Restricted maximum likelihood;GH: Growth hormone;SOD: Superoxide dismutase;GP: Glutathione peroxidase;DDCq: ΔΔCq

## Competing interests

The authors declare that they have no competing interests.

## Authors’ contributions

MFS, BOK, FN and KAG participated in the design and sampling of the study. MFS and BOK carried out the molecular studies and performed the statistical analysis. MFS, BOK, FN and KAG drafted the manuscript. All authors have read and approved the final manuscript.

## Supplementary Material

Additional file 1 AIC comparisons of the LME models. Click here for file

Additional file 2 Summary of linear mixed effect models testing for differences in log-weight and expression of the seven target genes in farmed versus hybrid salmon, hybrid versus wild salmon and wild versus farmed salmon. Click here for file

Additional file 3 **Cq values of the reference gene EF1A**_**A **_**and ΔΔCq values of the seven target genes in salmon of farmed, hybrid and wild origin, in both treatments.** Click here for file

Additional file 4 Linear regression between ΔΔDCq values on the y-axis and fish size (weight in grams) on the x-axis, for the seven selected genes, performed with a 95% confidence interval. Click here for file

Additional file 5 Linear regression between ΔΔDCq values on the y-axis and sampling period (1–3) on the x-axis, for the seven selected genes, performed with a 95% confidence interval. Click here for file

Additional file 6 Linear regression between ΔΔDCq values on the y-axis and time in anaesthesia (minutes) on the x-axis, for the seven selected genes, performed with a 95% confidence interval. Click here for file

Additional file 7 Full data set supporting the results of this article, with index. Click here for file
