# Supplementary figures and images for: Effects of environmental stress on mRNA expression levels of seven genes related to oxidative stress and growth in Atlantic salmon Salmo salar L. of farmed, hybrid and wild origin
Source: BMC Res Notes. 2012 Dec 5;5:672. doi: 10.1186/1756-0500-5-672 (PMC3598671; doi:10.1186/1756-0500-5-672)

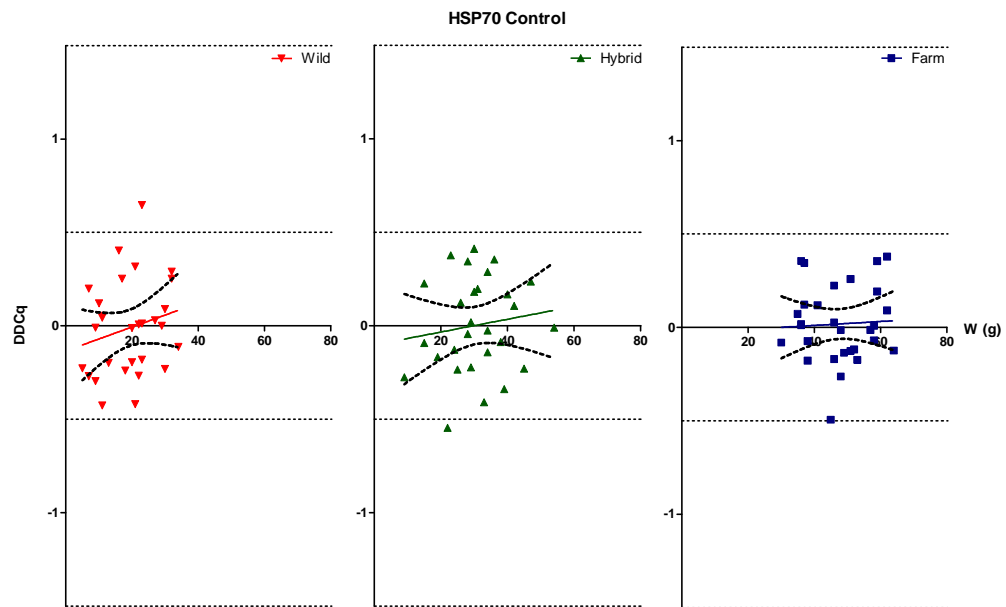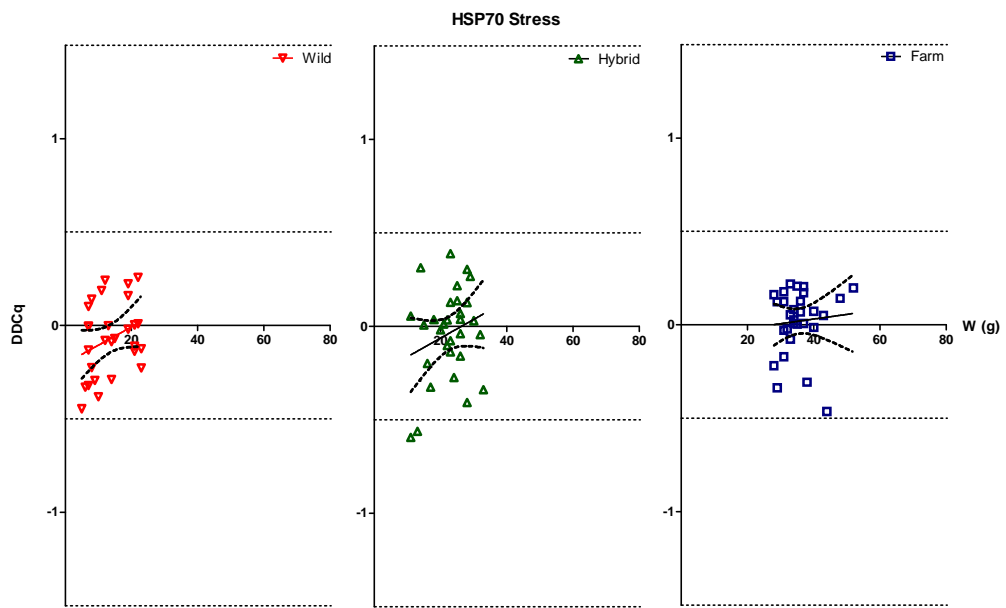

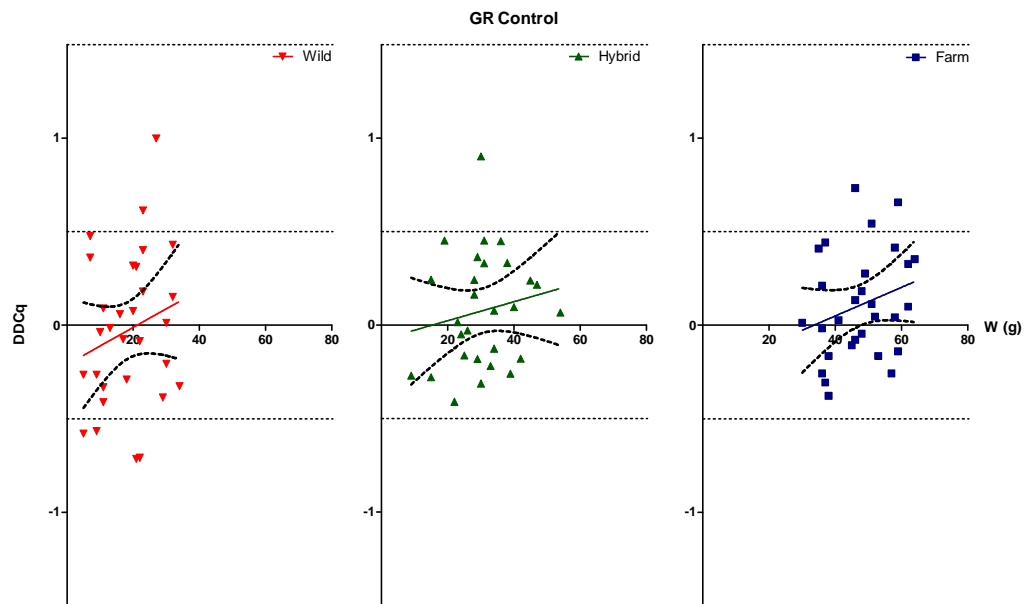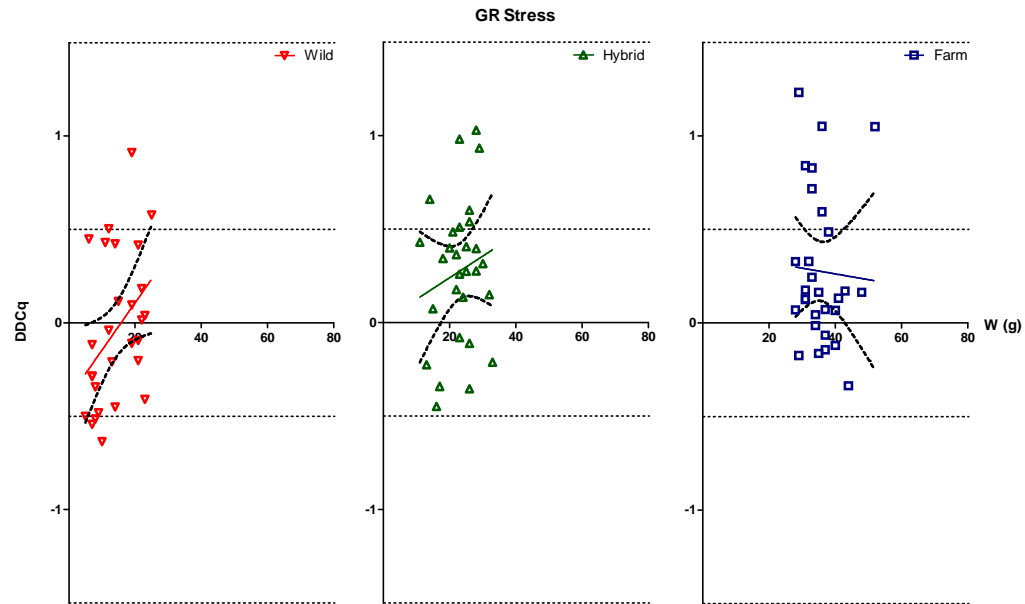

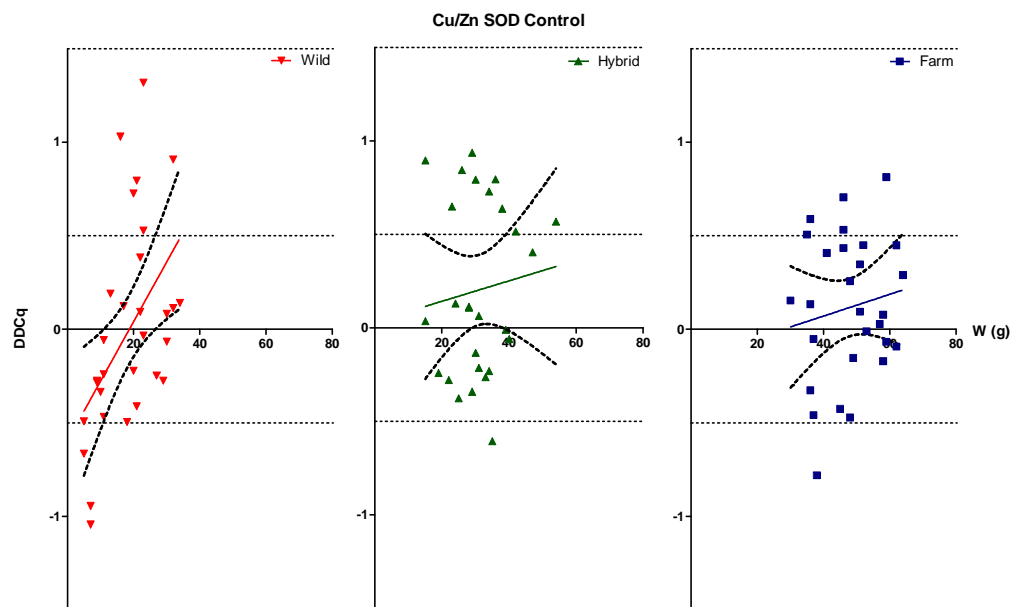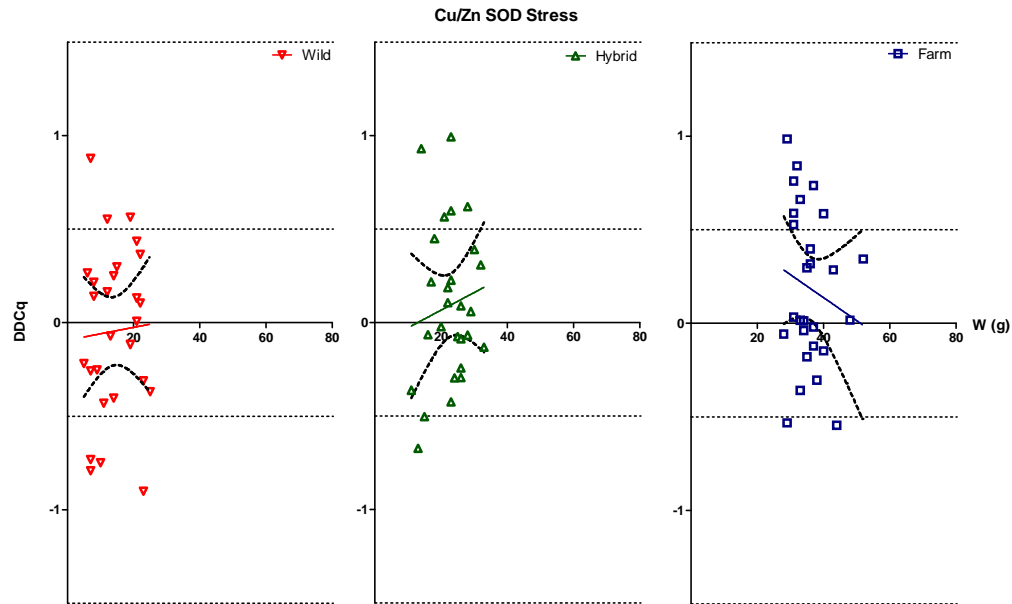

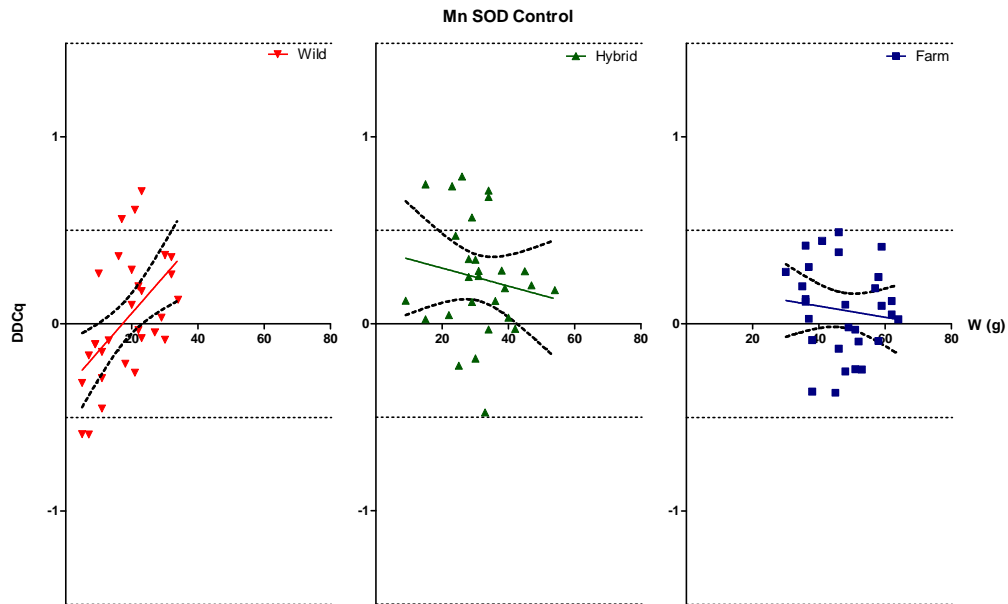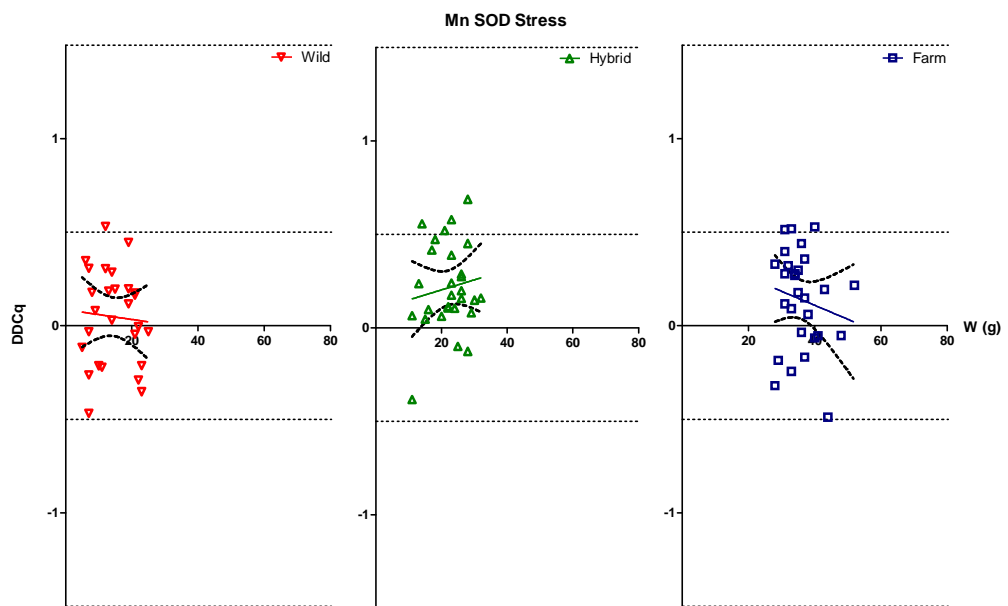

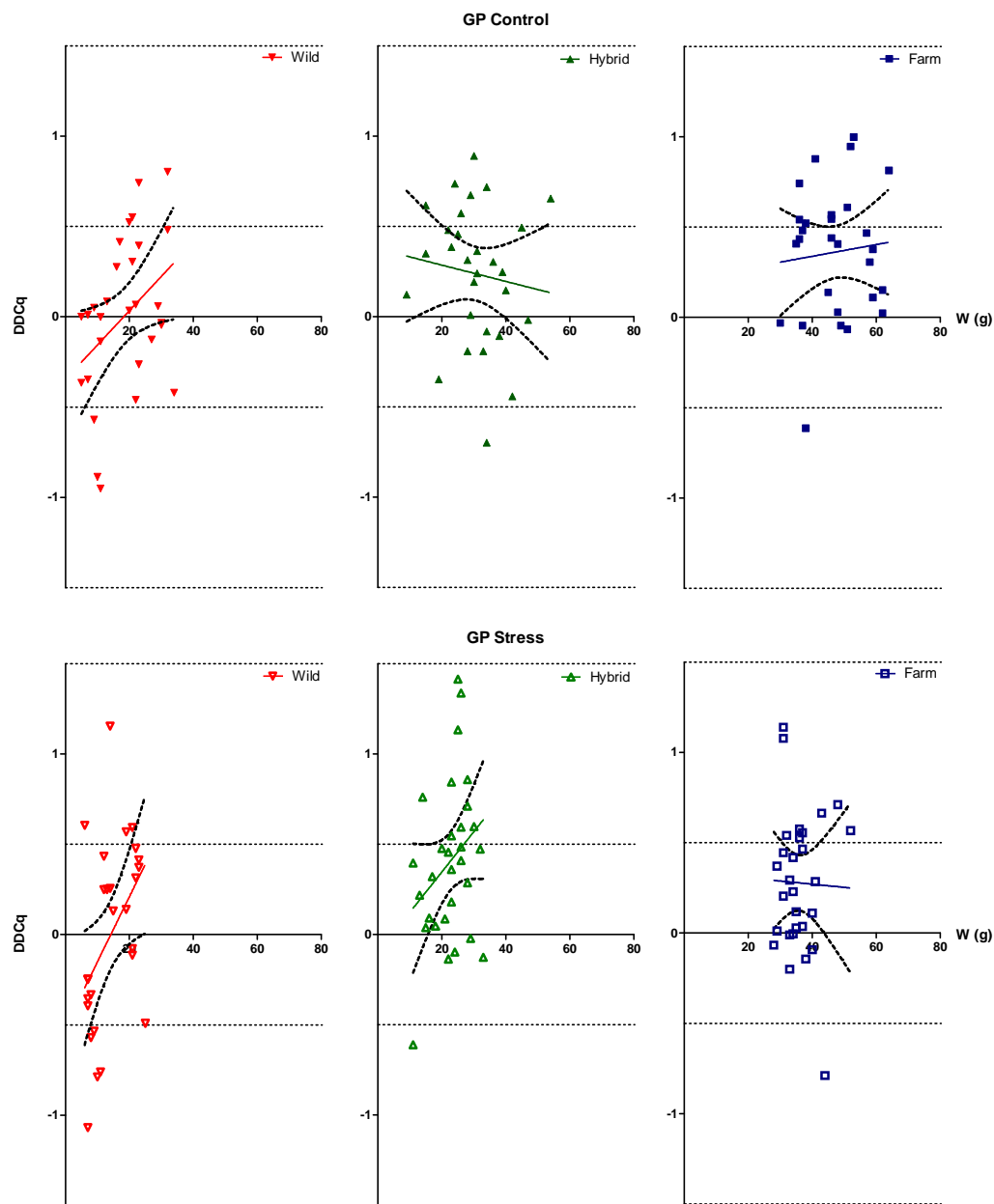

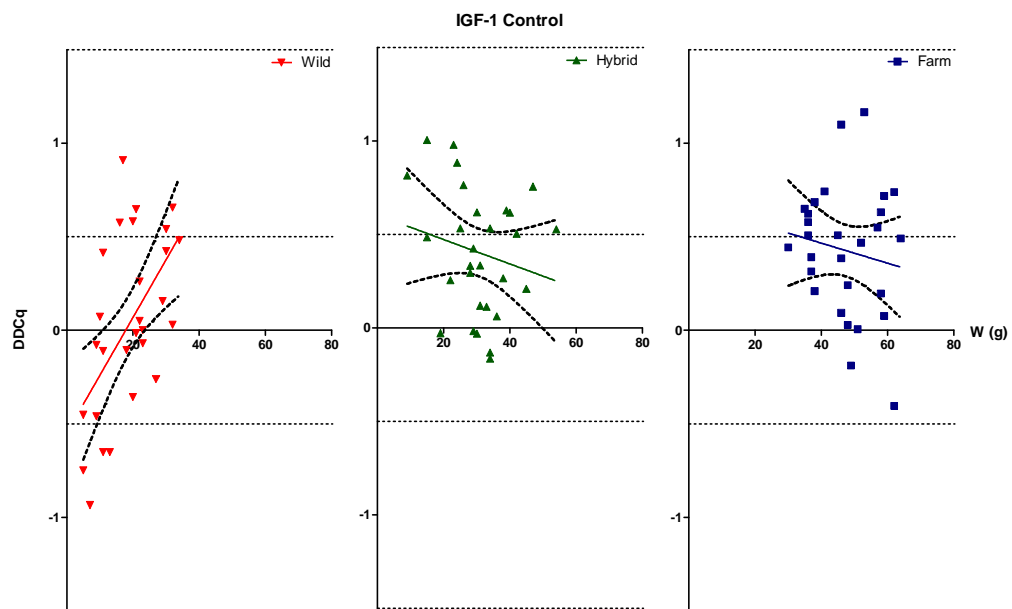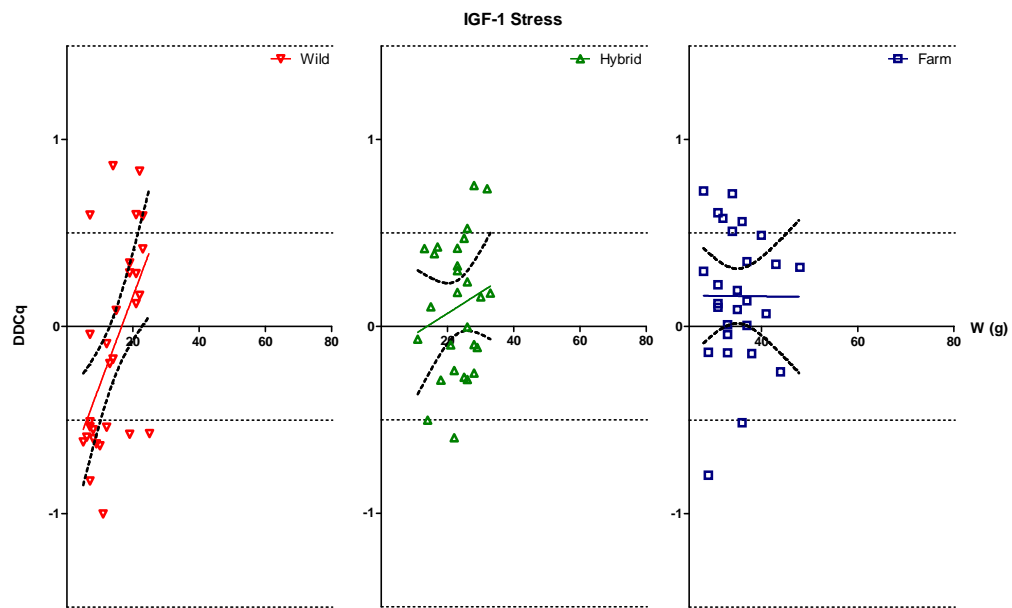

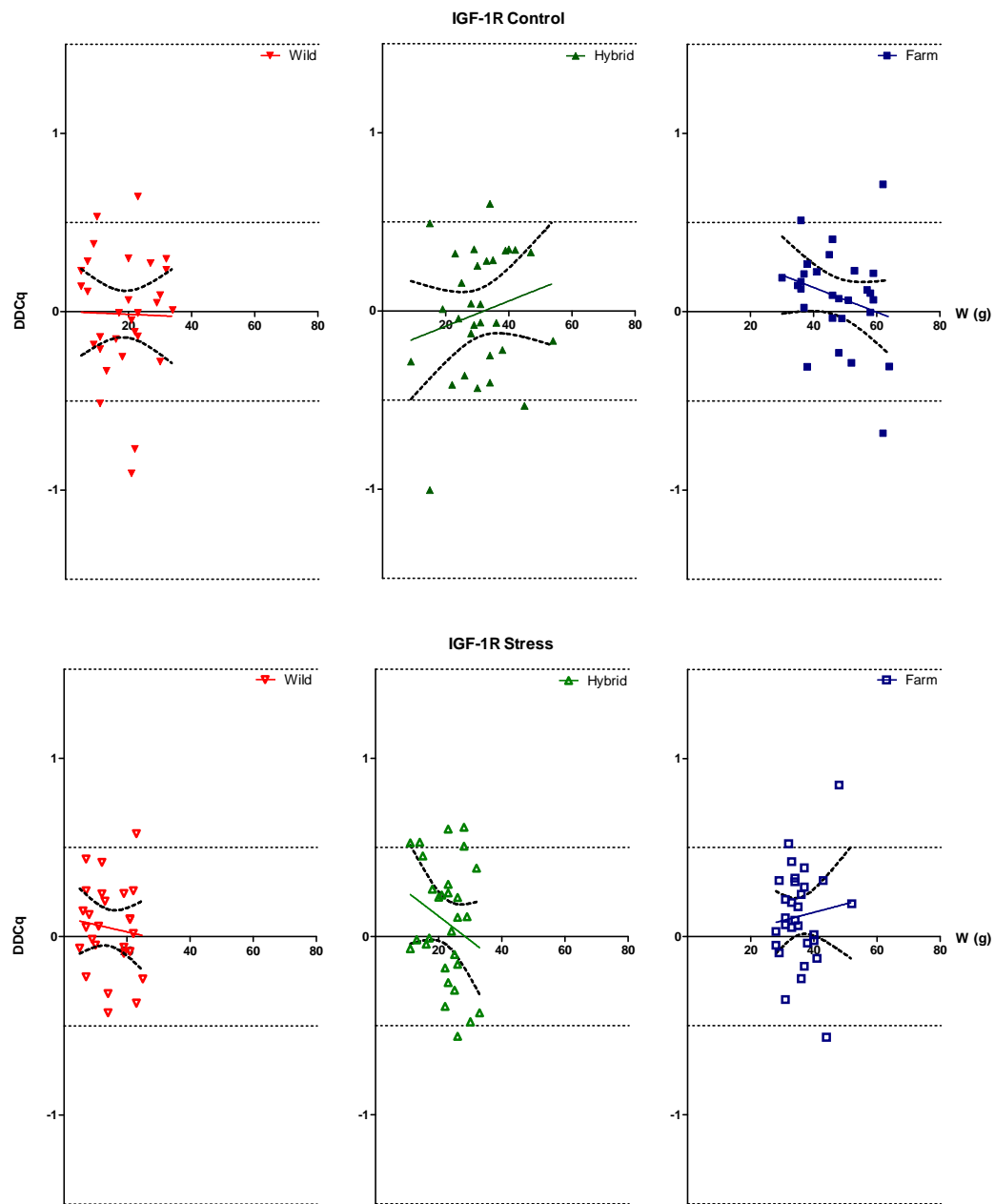

Supplement: Additional file 4 — Linear regression between ΔΔDCq values on the y-axis and fish size (weight in grams) on the x-axis, for the seven selected genes, performed with a 95% confidence interval. [file 1756-0500-5-672-S4.pdf]

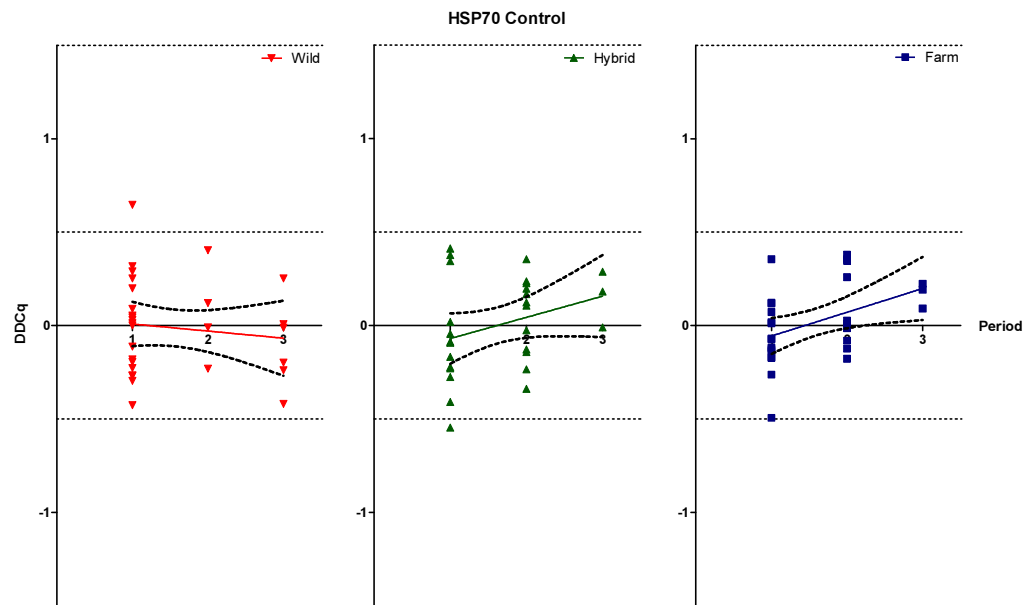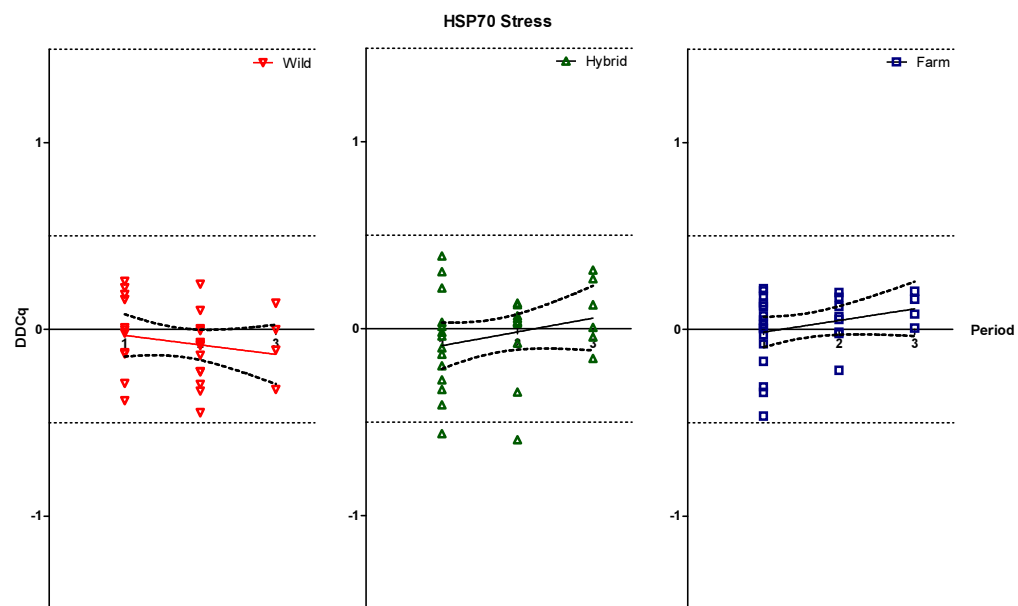

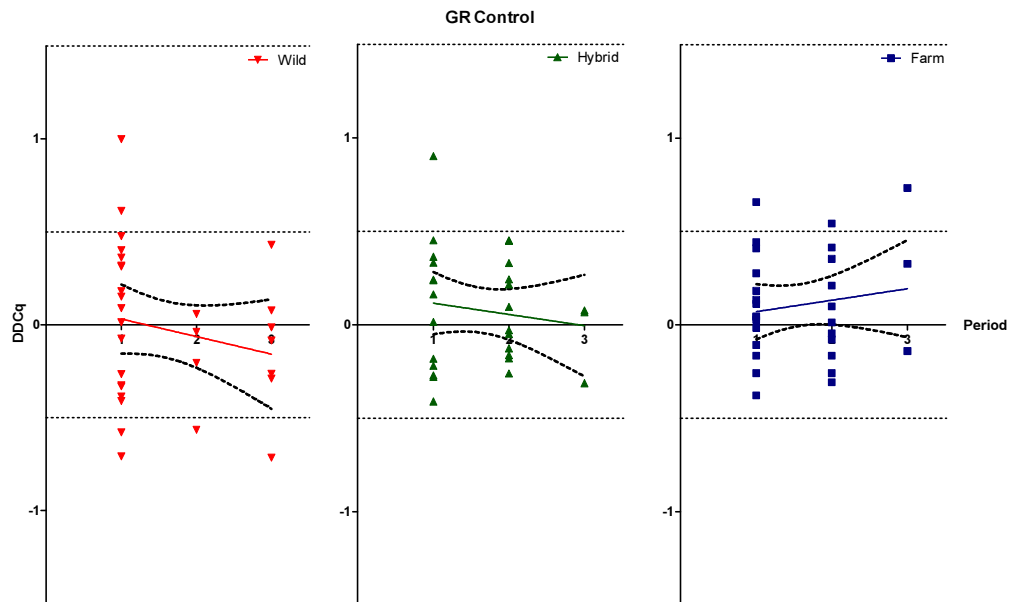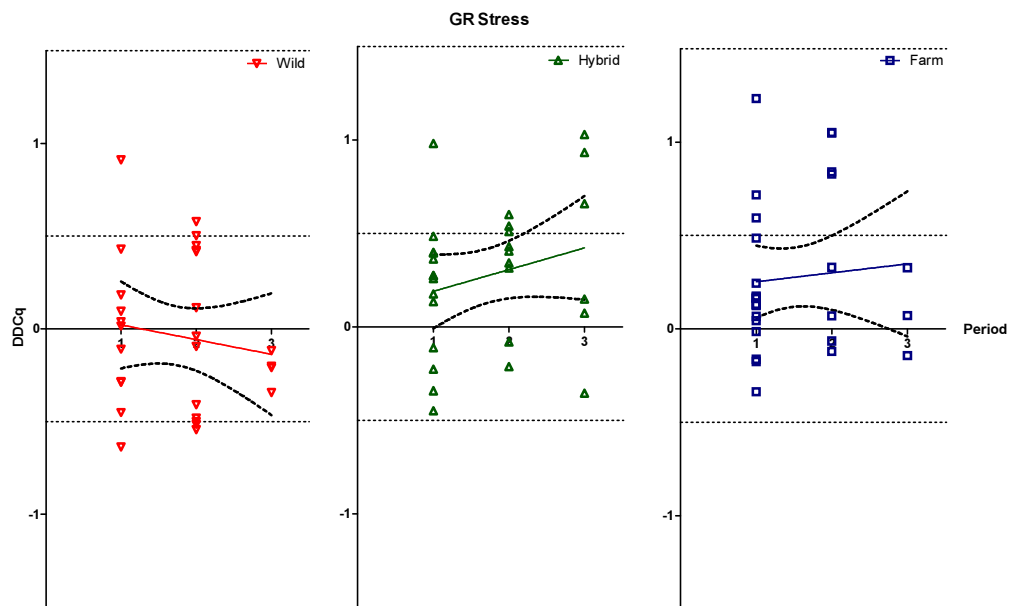

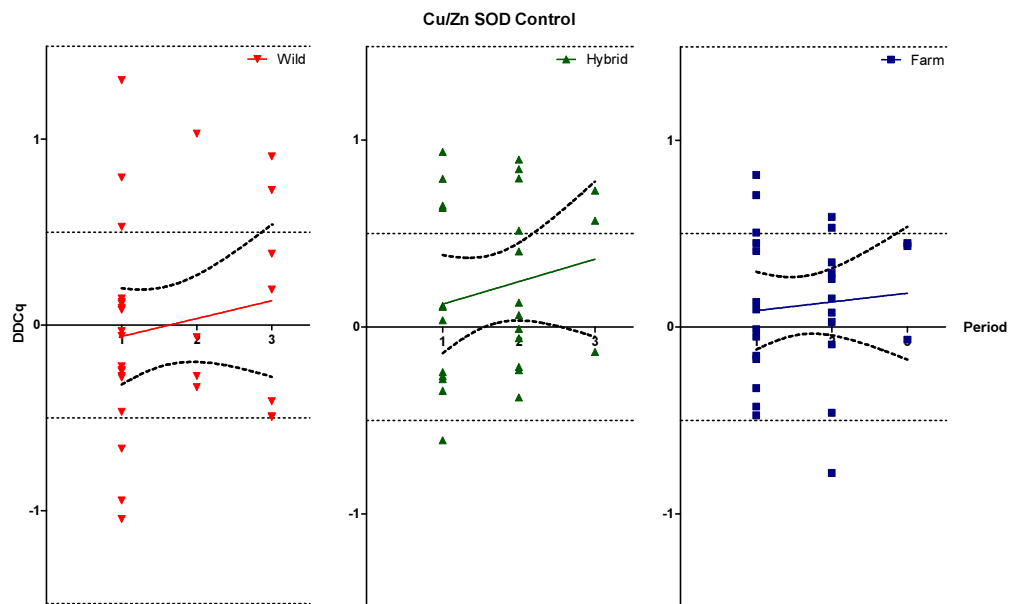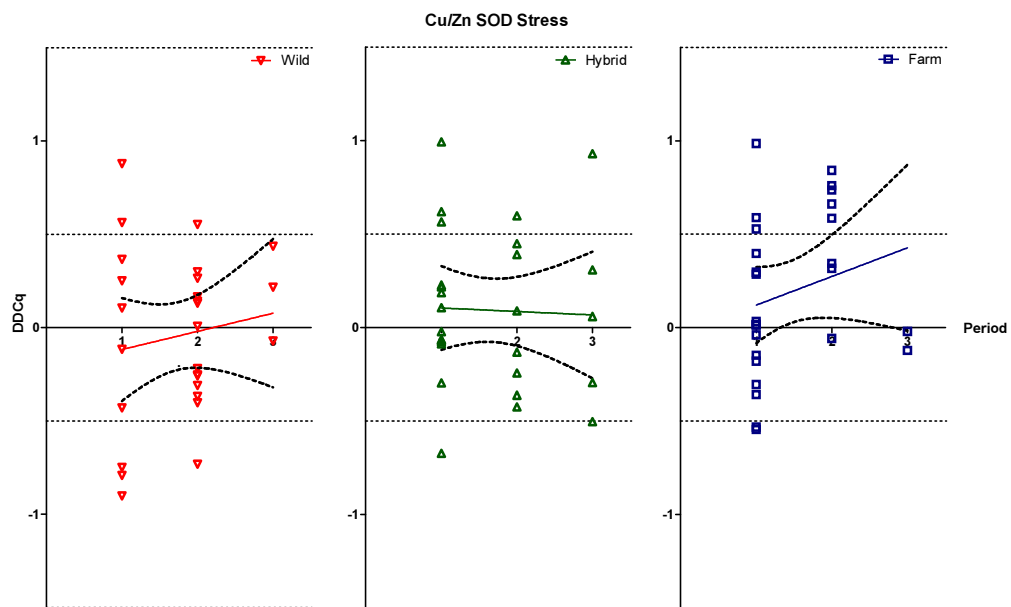

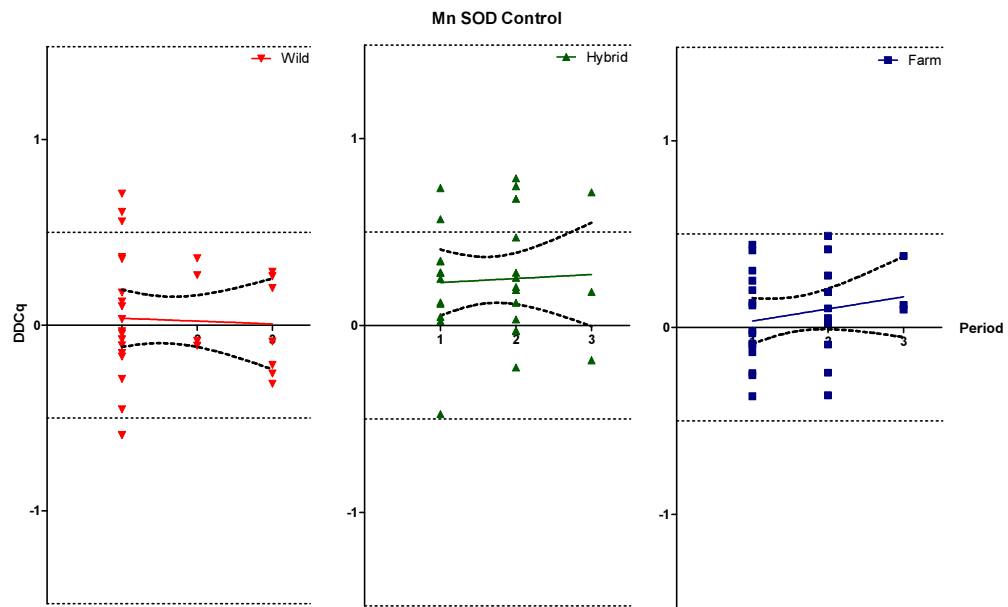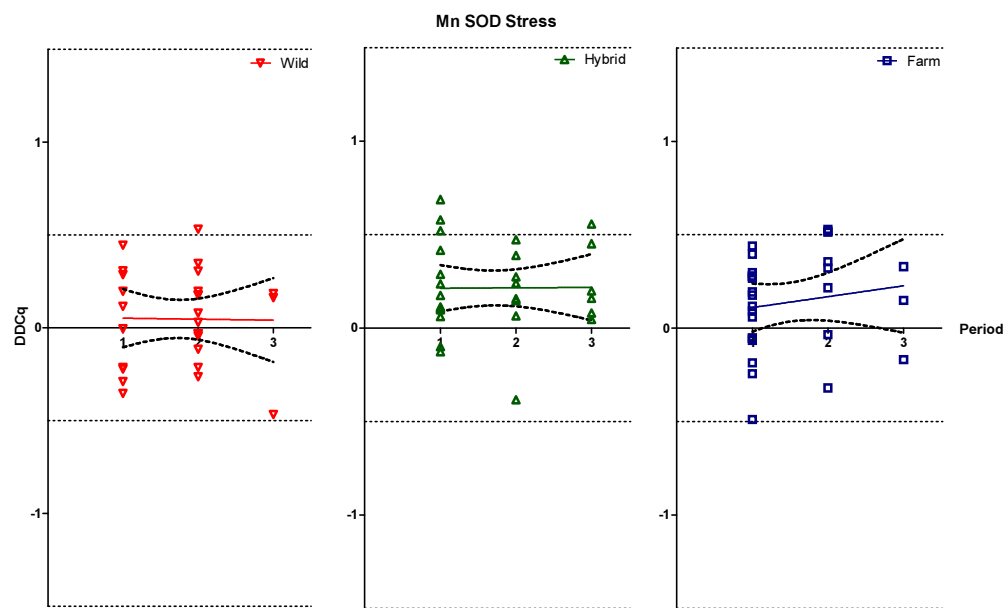

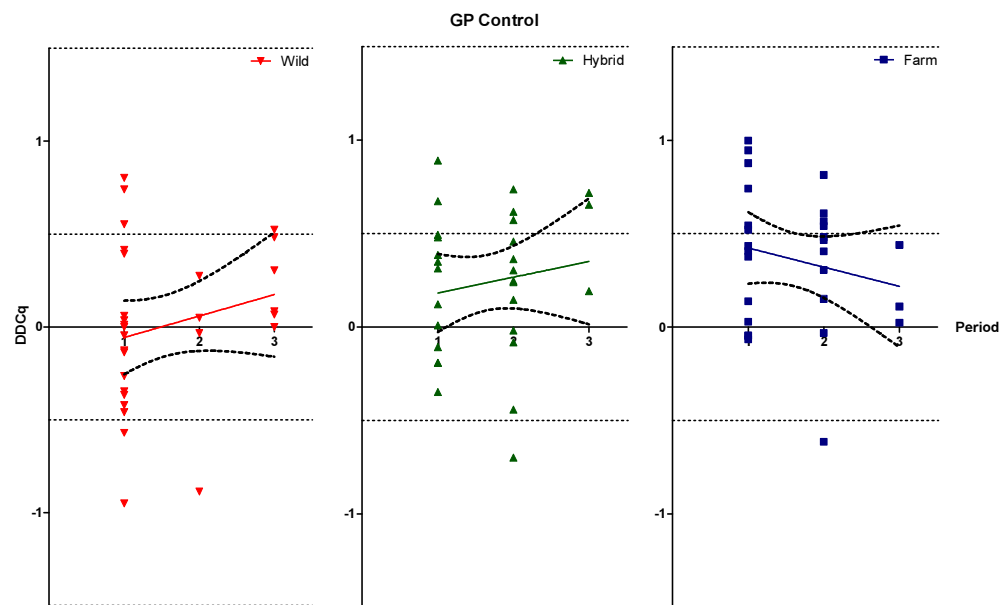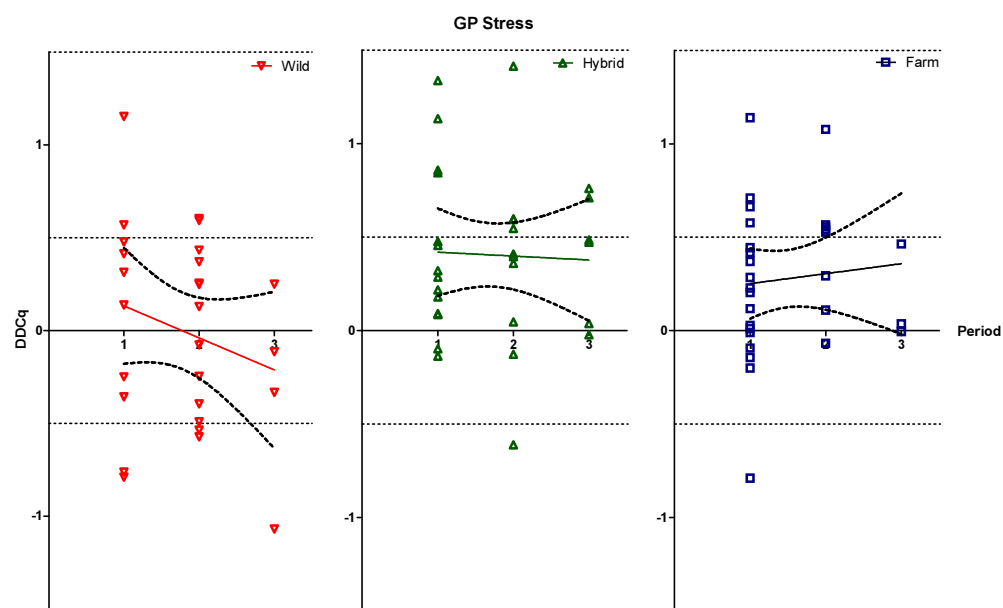

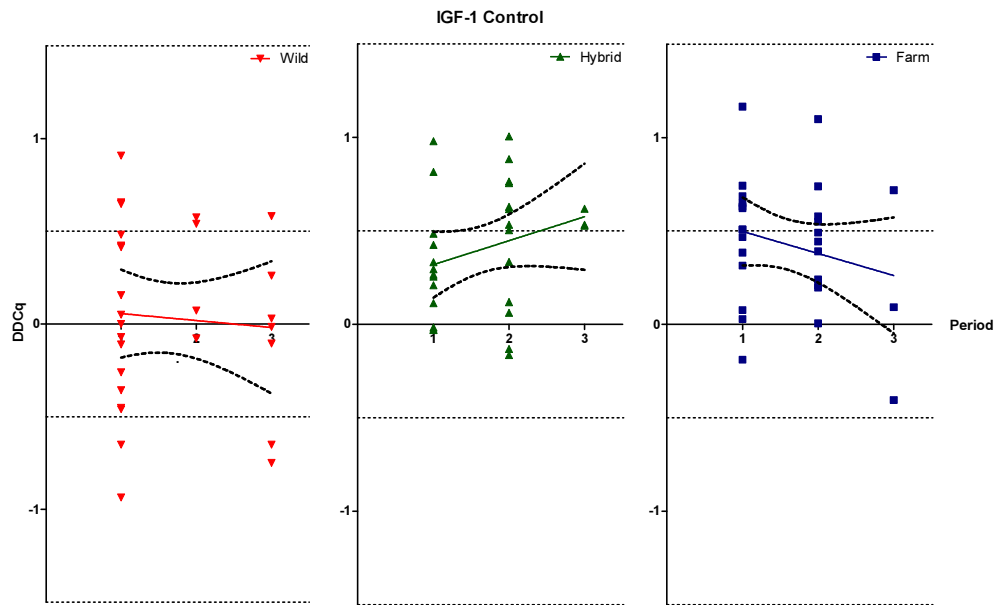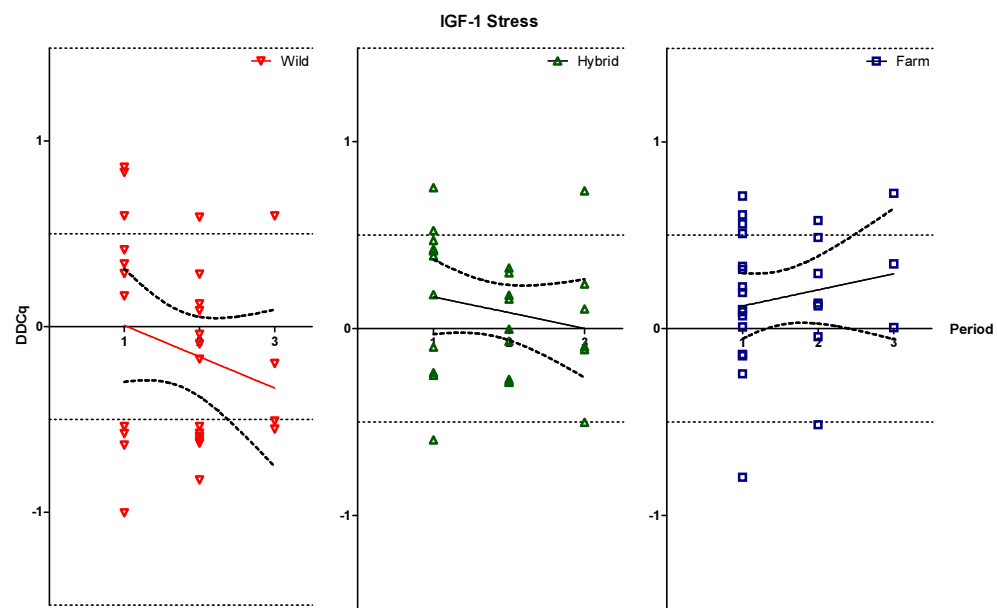

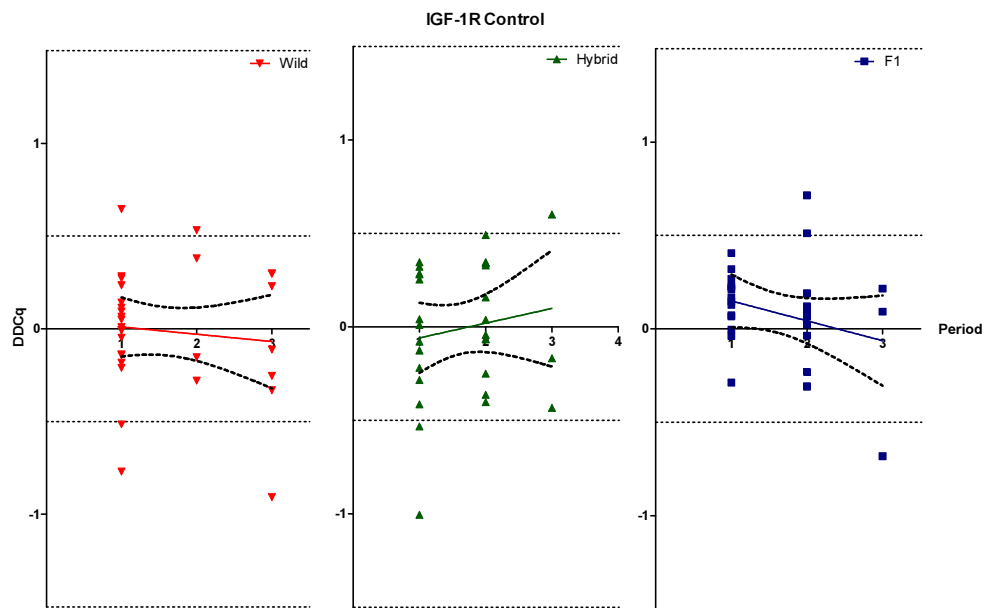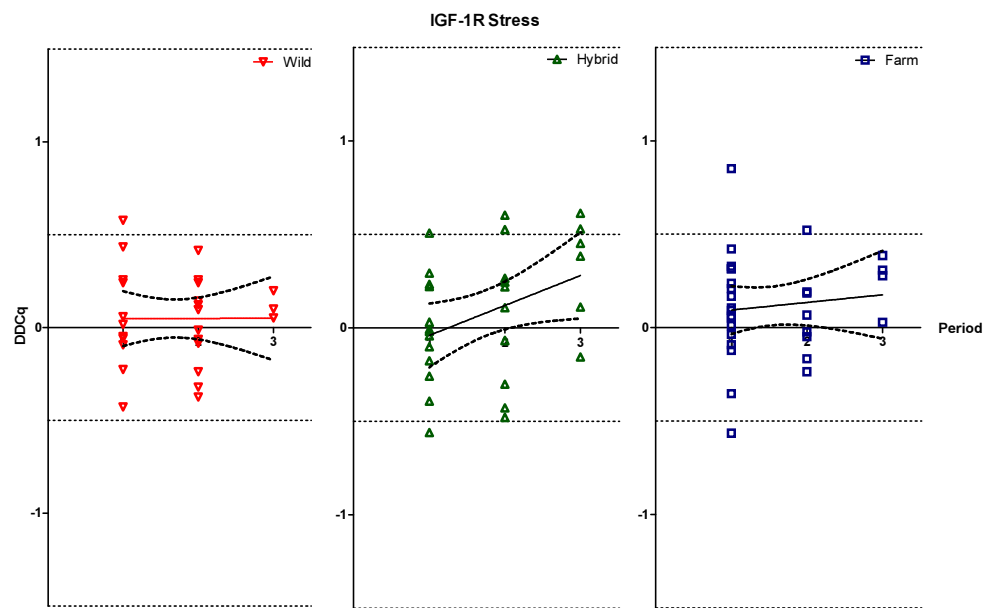

Supplement: Additional file 5 — Linear regression between ΔΔDCq values on the y-axis and sampling period (1–3) on the x-axis, for the seven selected genes, performed with a 95% confidence interval. [file 1756-0500-5-672-S5.pdf]

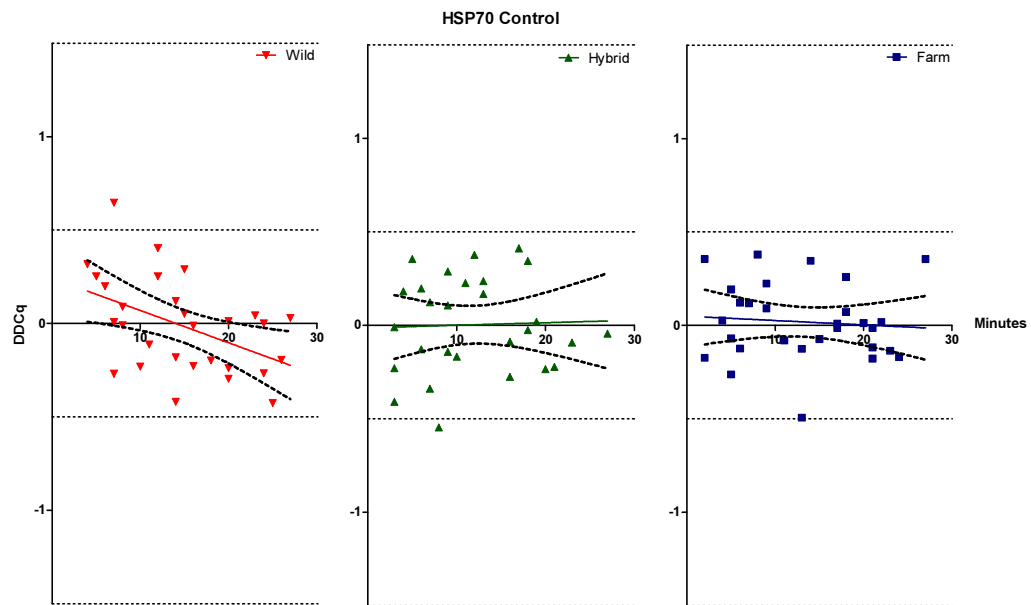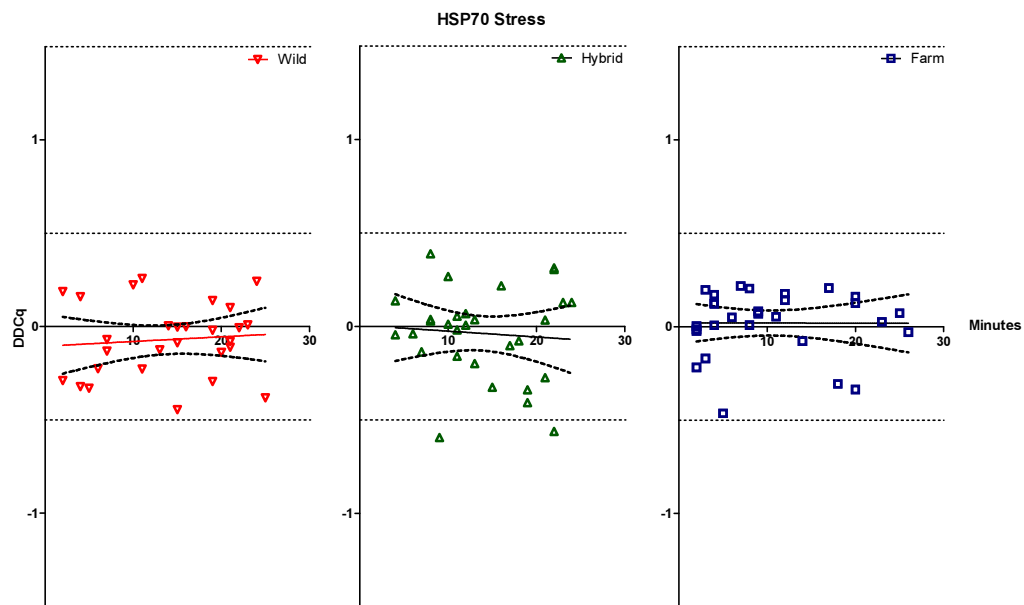

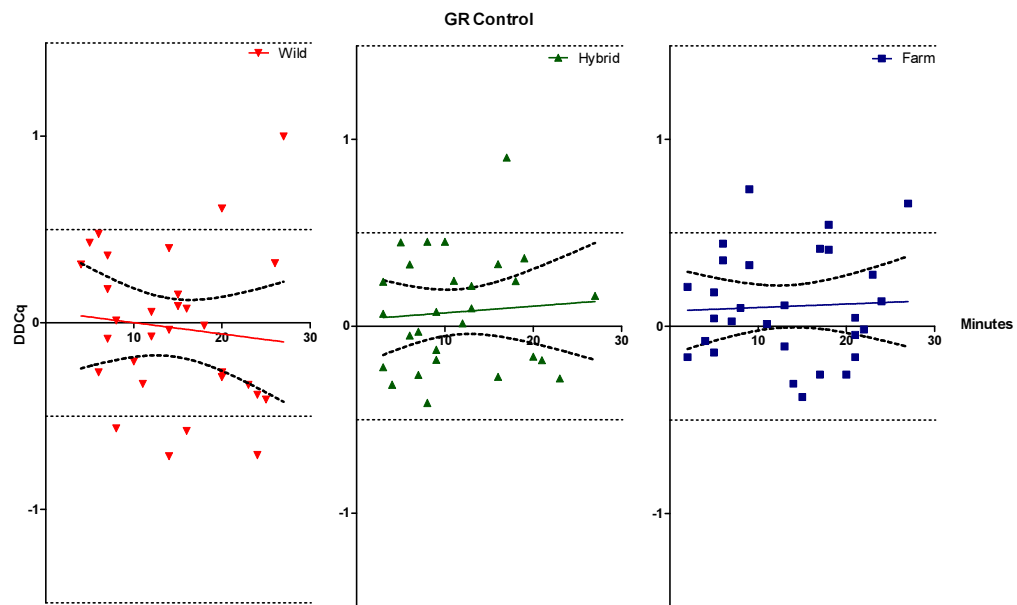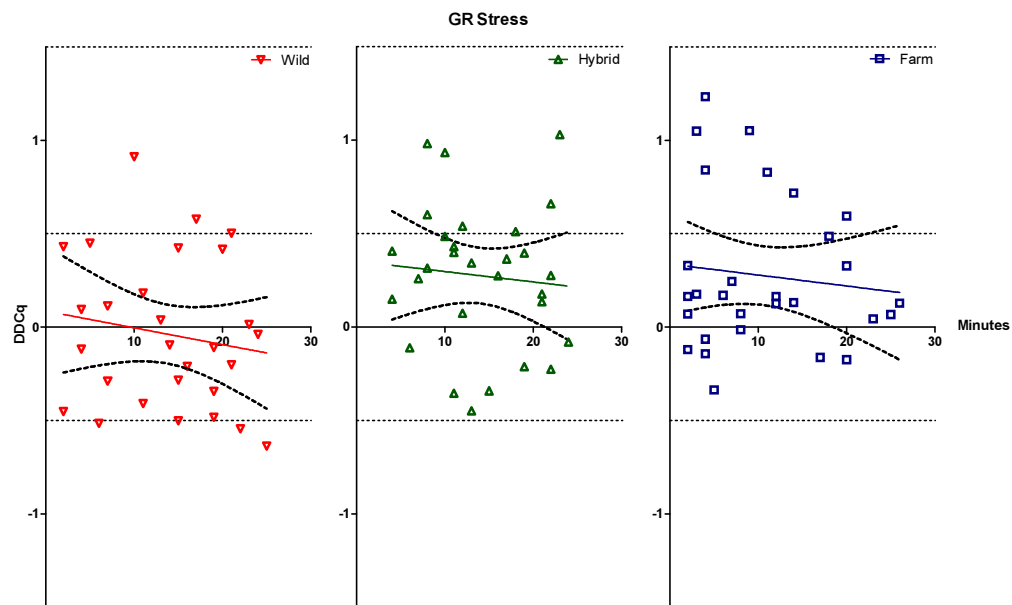

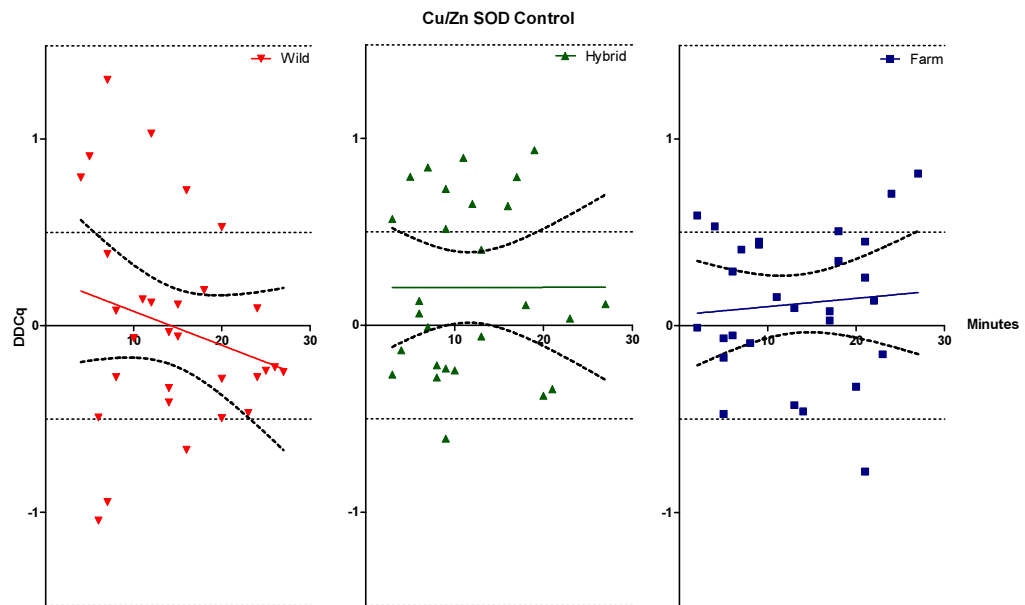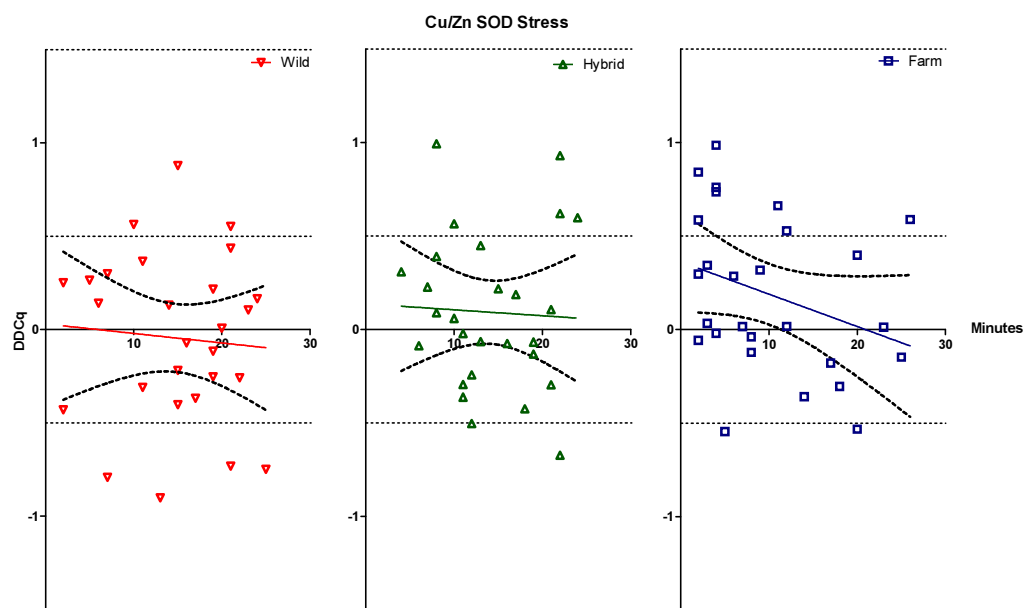

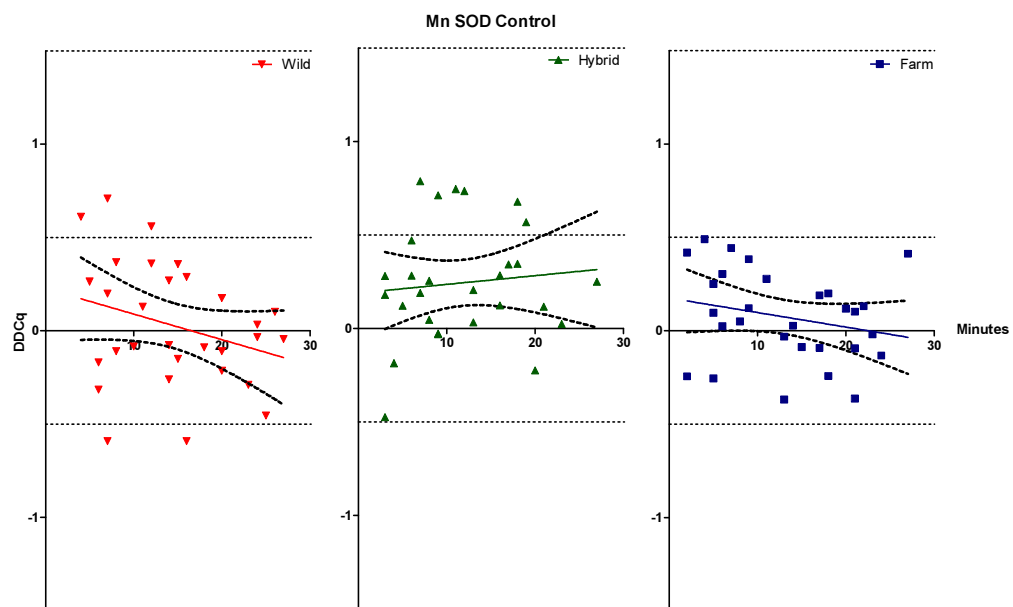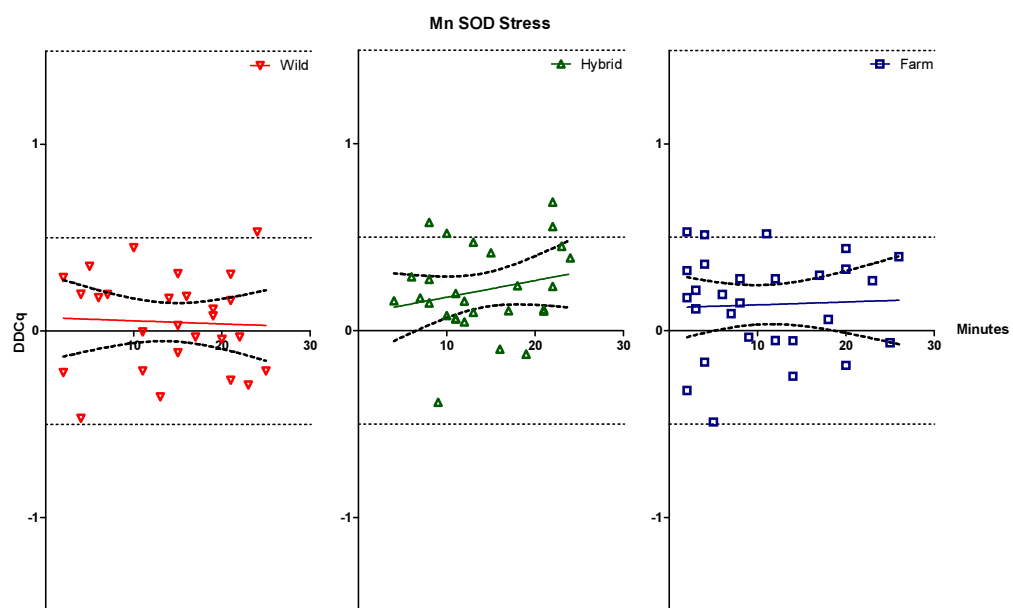

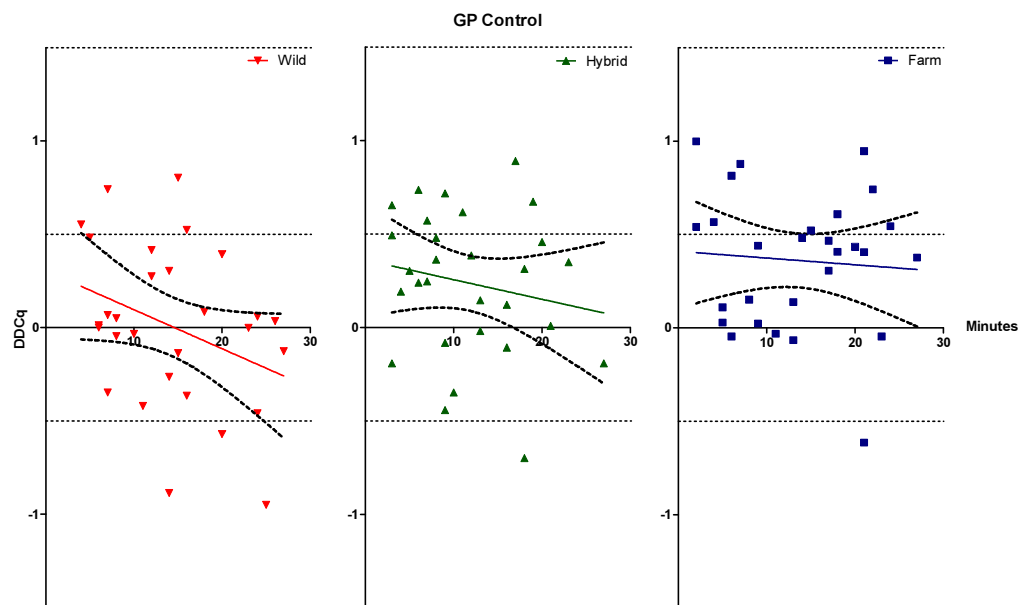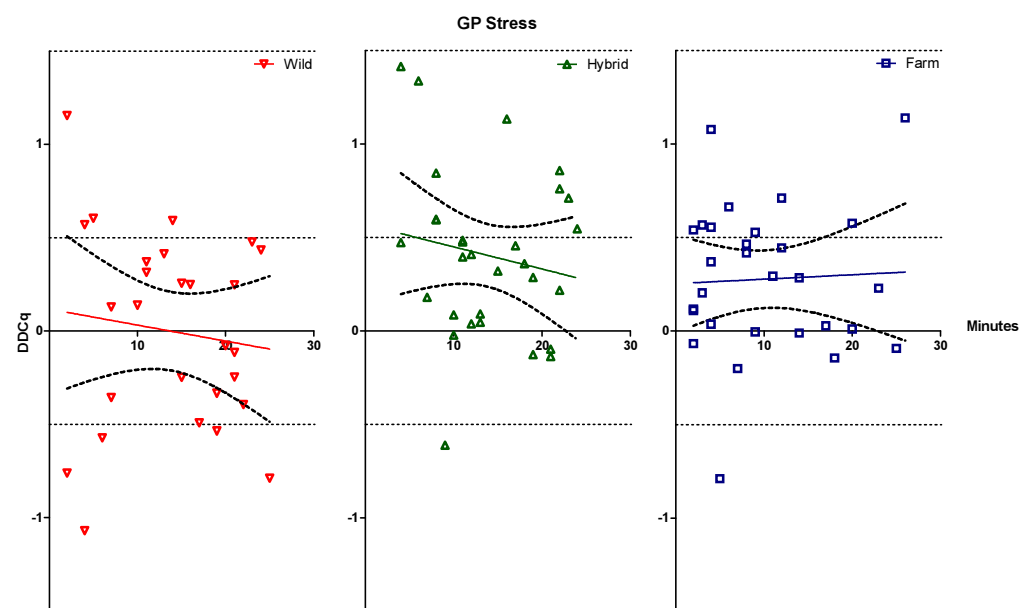

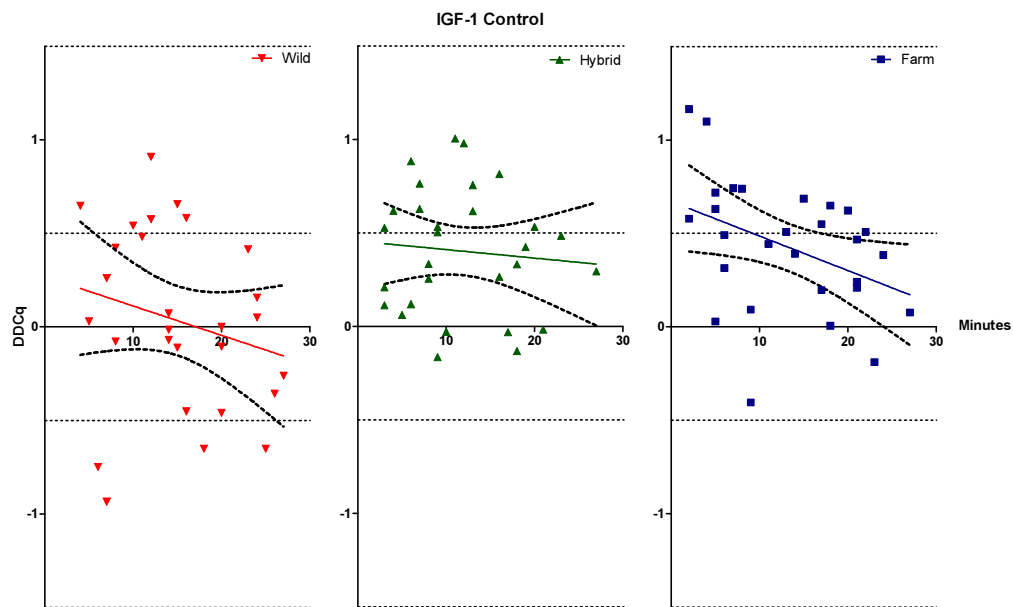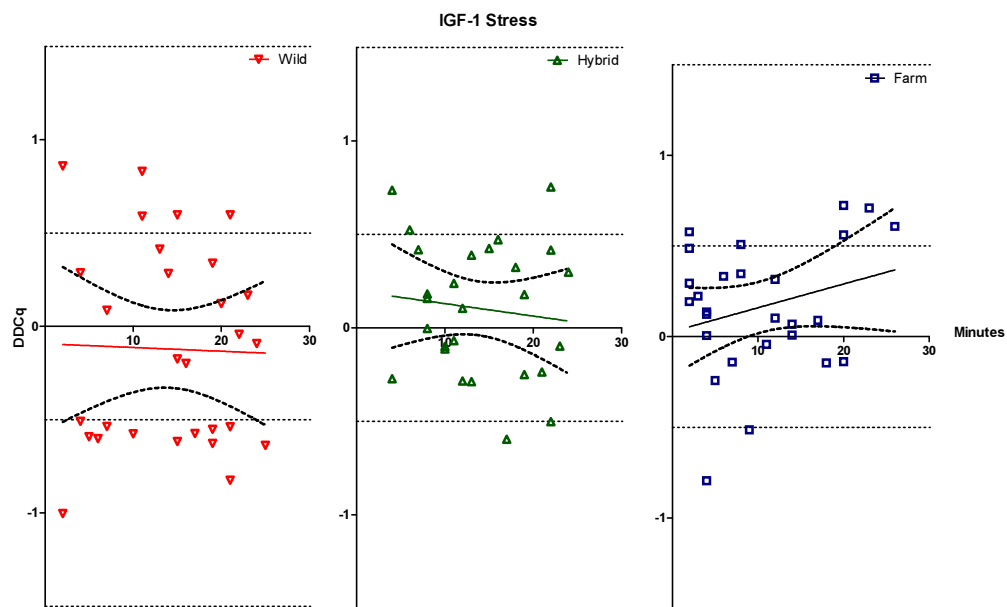

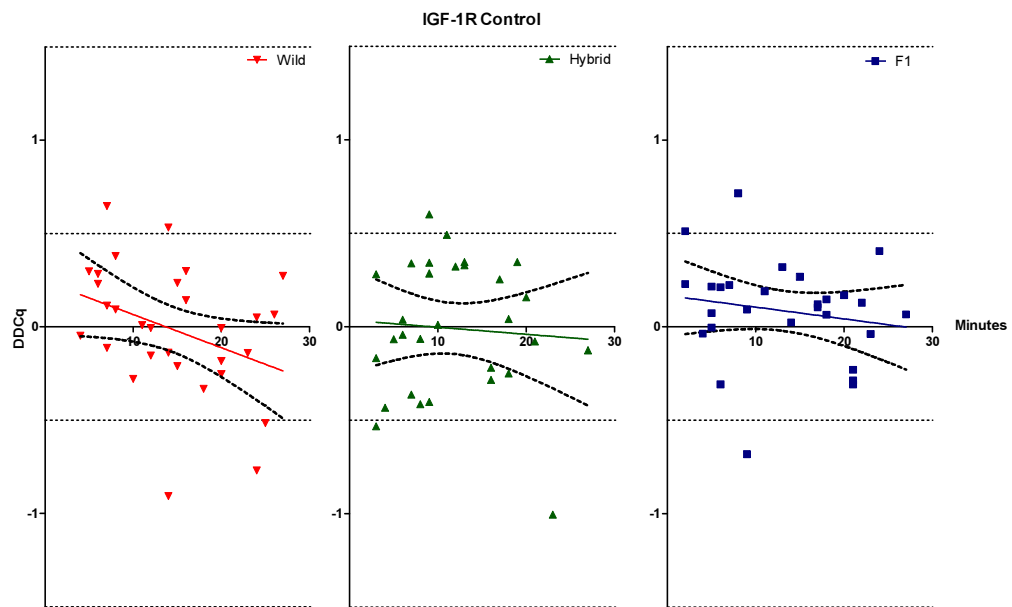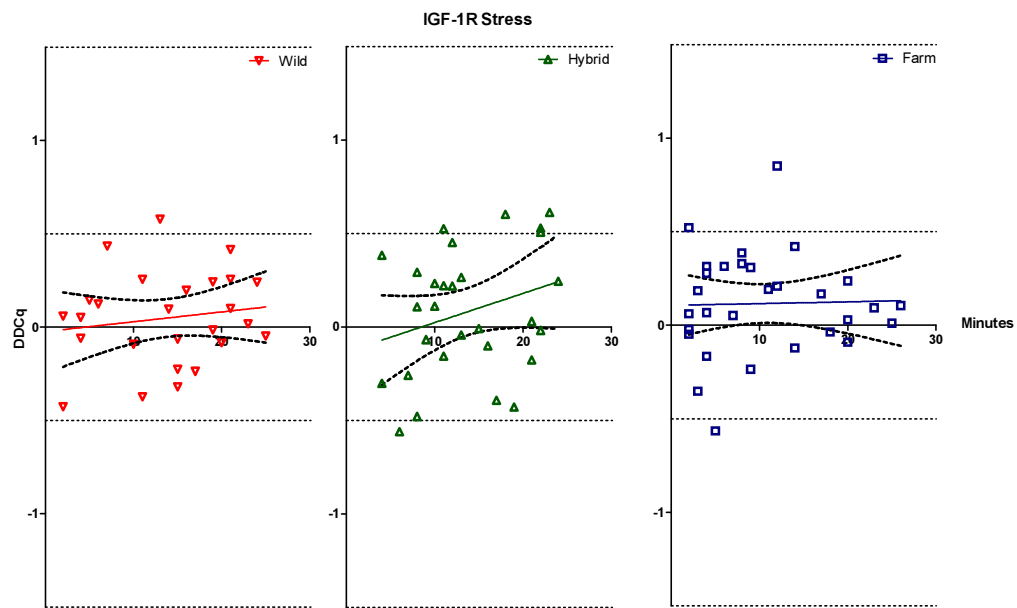

Supplement: Additional file 6 — Linear regression between ΔΔDCq values on the y-axis and time in anaesthesia (minutes) on the x-axis, for the seven selected genes, performed with a 95% confidence interval. [file 1756-0500-5-672-S6.pdf]
